# Immune-Related Genes for Predicting Future Kidney Graft Loss: A Study Based on GEO Database

**DOI:** 10.3389/fimmu.2022.859693

**Published:** 2022-02-25

**Authors:** Meng Dou, Chenguang Ding, Bingxuan Zheng, Ge Deng, Kun Zhu, Cuixiang Xu, Wujun Xue, Xiaoming Ding, Jin Zheng, Puxun Tian

**Affiliations:** ^1^ Department of Kidney Transplantation, Hospital of Nephropathy, The First Affiliated Hospital of Xi’an Jiaotong University, Xi’an, China; ^2^ Center of Shaanxi Provincial Clinical Laboratory, Shaanxi Provincial People’s Hospital, Xi’an, China

**Keywords:** immune-related genes, graft rejection, graft loss, prognostic model, kidney transplantation, immune infiltration

## Abstract

**Objective:**

We aimed to identify feature immune-related genes that correlated with graft rejection and to develop a prognostic model based on immune-related genes in kidney transplantation.

**Methods:**

Gene expression profiles were obtained from the GEO database. The GSE36059 dataset was used as a discovery cohort. Then, differential expression analysis and a machine learning method were performed to select feature immune-related genes. After that, univariate and multivariate Cox regression analyses were used to identify prognosis-related genes. A novel Riskscore model was built based on the results of multivariate regression. The levels of these feature genes were also confirmed in an independent single-cell dataset and other GEO datasets.

**Results:**

15 immune-related genes were expressed differently between non-rejection and rejection kidney allografts. Those differentially expressed immune-related genes (DE-IRGs) were mainly associated with immune-related biological processes and pathways. Subsequently, a 5-immune-gene signature was constructed and showed favorable predictive results in the GSE21374 dataset. Recipients were divided into the high-risk and low-risk groups according to the median value of RiskScore. The GO and KEGG analysis indicated that the differentially expressed genes (DEGs) between high-risk and low-risk groups were mainly involved in inflammatory pathways, chemokine-related pathways, and rejection-related pathways. Immune infiltration analysis demonstrated that RiskScore was potentially related to immune infiltration. Kaplan-Meier survival analysis suggested that recipients in the high-risk group had poor graft survival. AUC values of 1- and 3-year graft survival were 0.804 and 0.793, respectively.

**Conclusion:**

Our data suggest that this immune-related prognostic model had good sensitivity and specificity in predicting the 1- and 3-year kidney graft survival and might act as a useful tool for predicting kidney graft loss.

## Introduction

Kidney transplantation offers the optimal survival and quality of life for patients with end-stage renal disease (ESRD). More than 100, 000 new organ transplantations are performed annually worldwide (1). However, the existing immunosuppressive drugs are not enough in preventing kidney transplant recipients from the occurrence of graft rejection. Graft rejection is the main barrier to successful long-term transplantation (2, 3).

Implanted kidneys could be recognized as foreign by the recipient immune system. Then, the immune response is regulated by the innate and adaptive immune systems, which will result in the rejection of the transplanted kidneys. The immune response is a main cause of graft rejection and loss (4, 5). Immune-related genes have been involved in the regulation of innate trained/memory immunity. Post-transplantation expression of immune-related genes could reflect the immune status of recipients and allograft rejection is closely associated with immune-related molecular changes in renal allograft tissues. Immune-related molecular changes in kidney allografts would reflect the processes that lead to graft loss. However, it is still unclear how immune-related genes participate in allograft rejection and whether they can be applied as prognostic markers for graft loss. Since immune-related genes play essential roles in allograft rejection, the use of immune-related genes for prognosis estimation was highlighted in many diseases (6–8). We hypothesized that immune-related genes may predict kidney graft loss.

In the present study, one goal was to explore the relationship between immune-related genes expression and graft rejection in kidney allografts and the other was to evaluate the predictive value of immune-related genes for kidney graft loss. Gene expression profiles of kidney allografts were obtained from the GEO database. The expression and potential molecular mechanism of immune-related genes in rejection samples were explored. Finally, an immune-gene signature was built to predict graft loss after kidney transplantation.

## Materials and Methods

### Data Collection and Processing

Bulk RNA-seq and microarray expression datasets used in this study were obtained from the GEO database (https://www.ncbi.nlm.nih.gov/geo/). Totally, 8 datasets were collected. The information of these datasets is shown in **Table 1**. All microarray datasets were subjected to log2 transformation and normalized using the R package “limma”. RNA sequencing datasets were transformed into a log2(TPM + 1) scale. Single-cell transcriptomes of one rejection kidney sample were downloaded and visualized using the Kidney Integrative Transcriptomics (K.I.T.) database (http://humphreyslab.com/SingleCell/).

**Table 1:** Description of datasets in this study.

Accession	Platform	Species	Tissues	References (PMID)
GSE36059	GPL570	Homo sapiens	Kidney	24700874
GSE48581	GPL570	Homo sapiens	Kidney	25377077
GSE50058	GPL570	Homo sapiens	Kidney	24127489
GSE75693	GPL570	Homo sapiens	Kidney	27165815
GSE72925	GPL570	Homo sapiens	Kidney	31632976
GSE131179	Illumina HiSeq 2500	Homo sapiens	Kidney	32102984
GSE9493	GPL570	Homo sapiens	Kidney	19191772
GSE21374	GPL570	Homo sapiens	Kidney	20501945

### Differential Immune-Related Genes Analysis

The list of human immune-related genes was obtained from the ImmPort database (9). We used R statistical software (version 4.0.3) and “limma” package to perform significance analysis of DE-IRGs between non-rejection and rejection renal tissues in the GSE36059 dataset. The adjusted p-value <0.05 and transcripts with a fold expression greater than 2 were used as the cutoff for difference analysis.

### Gene Ontology and KEGG Pathway Analysis

Gene ontology (GO) and Kyoto Encyclopedia of Genes and Genomes (KEGG) pathway enrichment analyses were performed to explore the functions and pathways of differentially expressed genes using the “clusterProfiler” package (10). A value of P < 0.05 was considered to be statistically significant.

### Construction of the Prognostic Gene Signature

Support vector machine (SVM) is a supervised learning method with very high accuracy and precision. The recursive feature elimination (RFE) algorithm yields much better classification compared to many other algorithms (11). Therefore, a method combining SVM with RFE was used for gene selection among all DE-IRGs. Then, univariate and multiple Cox regression analysis were performed in the GSE21374 dataset. P<0.05 was considered to be significant. After that, an immune-related prognostic signature was built based on multiple Cox regression analysis. The RiskScore of each recipient was obtained according to the following formula: $RiskScore=\Sigma_{i=1}^{n}\;\beta_{i}*Exp_{i}$ . Patients were divided into the high-risk and low-risk groups according to the median value of RiskScore in the GSE21374 dataset. The R package “survival” was used to perform Kaplan-Meier (KM) survival analysis between the two groups. In addition, the predictive value of RiskScore was evaluated through time-dependent receiver operating characteristic analyses using the R package “pROC”.

### Immune Infiltration Analysis

A bioinformatics algorithm called CIBERSORT was applied to assess the level of immune infiltration in selected samples. Immune cell proportions in the kidney allografts with significant enrichment were acquired and reported in bar plots. We used the R package “vioplot” to compare the levels of each immune cell type. Spearman’s rank correlation analysis was performed to explore the correlations between infiltrated immune cells and feature genes or RiskScore. The R package “ggplot2” was used to visualize the plot.

### Gene Set Enrichment Analysis (GSEA)

Patients were divided into the high-risk and low-risk groups according to the median value of RiskScore in the GSE21374 dataset. Then, the R package “limma” was used to compare differentially expressed genes between the high-risk and low-risk groups. We used the R package “clusterProfiler” to perform the Gene Set Enrichment Analysis.

### Statistical Analysis

All statistical analyses were conducted using R software (version 4.0.3). Student’s t-test for normally distributed variables and Mann-Whitney U test for abnormally distributed variables were conducted to compare the differences between two groups. The value of p < 0.05 was regarded statistically significant. The “pheatmap” package was used to conduct hierarchical clustering analysis. ROC analysis was performed using the “pROC” package, and the area under the curve (AUC) was calculated.

## Results

### Screening of DE-IRGs and Functional Analysis

**Figure 1:**
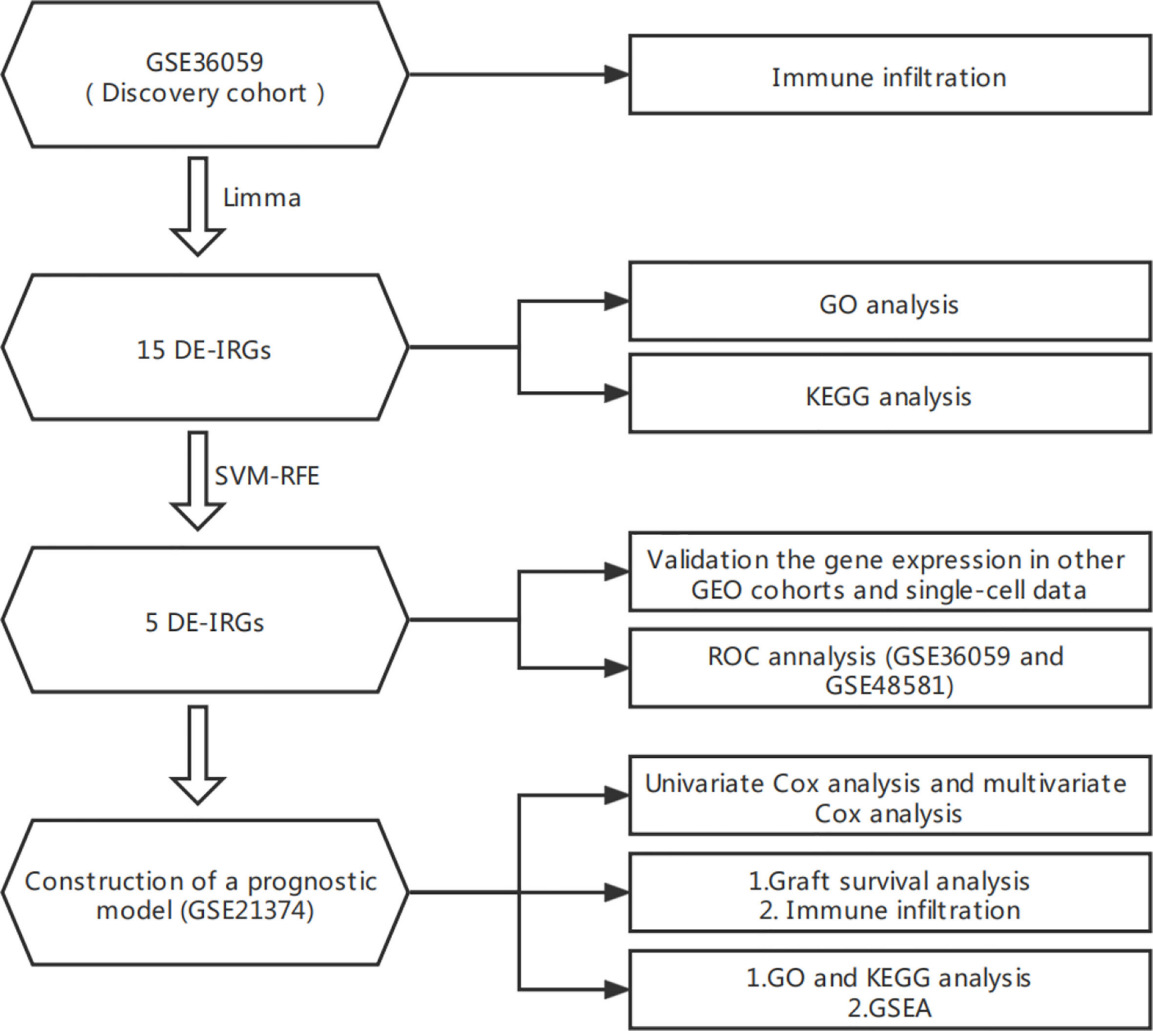
Flowchart of this research.

**Figure 2:**
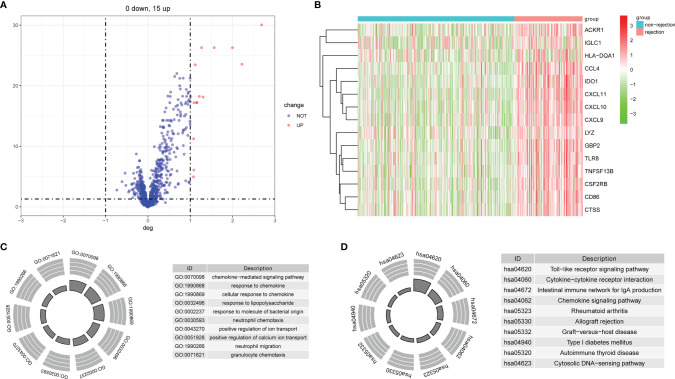
Identification of DE-IRGs and enrichment analysis. **(A)** Differentially expressed immune-related genes between rejection and non-rejection samples. **(B)** The heat map of DE-IRGs in the discovery cohort. **(C)** GO functional enrichment analyses. **(D)** KEGG functional enrichment analyses.

The flowchart of this study is shown in **Figure 1**. All the GEO datasets used in this research are summarized in **Table 1**. The GSE36059 dataset was used as a discovery cohort to screen DE-IRGs. A total of 15 DE-IRGs were identified between non-rejection and rejection tissues (**Figure 2A**). All differentially expressed genes were upregulated (**Figure 2B**). GO enrichment and KEGG pathway analyses were performed to study the functional roles of the DE-IRGs (**Figures 2C, D**). Based on our results, immune-related biological processes and pathways were significantly enriched.

### Validation of Gene Expression

**Figure 3:**
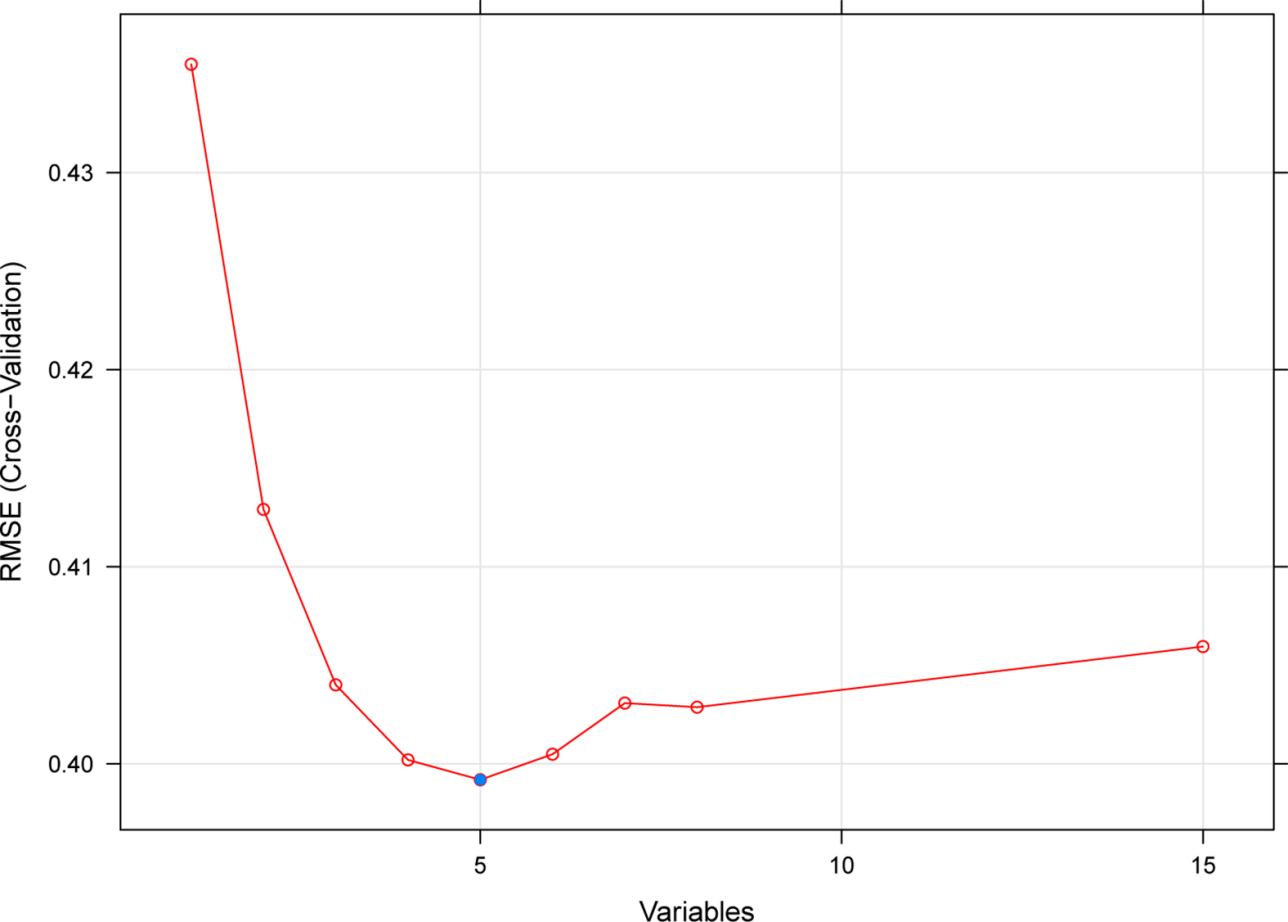
A plot of feature immune-related gene selection by recursive feature elimination. The blue dot indicates the best five genes.

**Figure 4:**
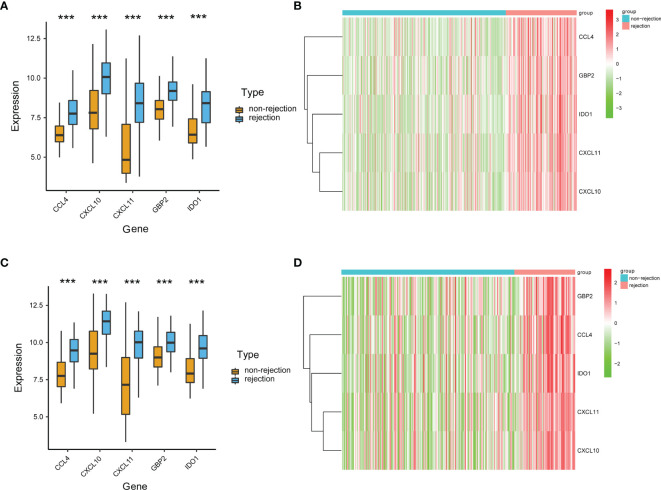
Validation of the expression levels of identified immune-related genes. **(A)** The expression levels of the identified five genes in the discovery cohort. **(B)** The heat map of identified genes in the discovery cohort. **(C)** The expression levels of the identified five genes in the GSE48581 dataset. **(D)** The heat map of identified genes in the GSE48581 dataset. ***p < 0.001.

Based on the SVM-RFE algorithm, 5 genes (CXCL11, CCL4, CXCL10, IDO1, and GBP2) were selected (**Figure 3**). In the GSE36059 dataset, the expression levels of 5 feature genes in rejection tissues were significantly higher than those in non-rejection tissues (**Figure 4A**). In the GSE48581 dataset, the expression levels of 5 feature genes in rejection tissues were also significantly upregulated (**Figure 4C**). Hierarchical clustering analysis in the GSE36059 and GSE48581 datasets showed that samples were clearly separated into two clusters based on the 5 identified genes (**Figures 4B, D**). To confirm feature gene expression changes in rejection samples, some additional independent GEO datasets were used to assess the expression of 5 immune-related genes. These results were similar to our previous results. The expression levels of 5 feature genes were also explored in GSE50058, GSE75693, GSE72925, GSE131179, and GSE9493 datasets. Their expression levels were significantly elevated in the rejection tissues (See **File S1**). The expression of these feature genes was also explored at the single-cell level (See **File S2**). We found that they have distinct expression patterns in rejection tissues. CXCL11 was mainly expressed in endothelial cells and cycling cells. CXCL10 was mainly expressed in monocytes, endothelial cells, and cycling cells. CCL4 was mainly expressed in monocytes, T cells, and mast cells. IDO1was mainly expressed in B cells. GBP2 was mainly expressed in immune cells.

### Diagnostic Values of Immune-Related Genes in Graft Rejection

**Figure 5:**
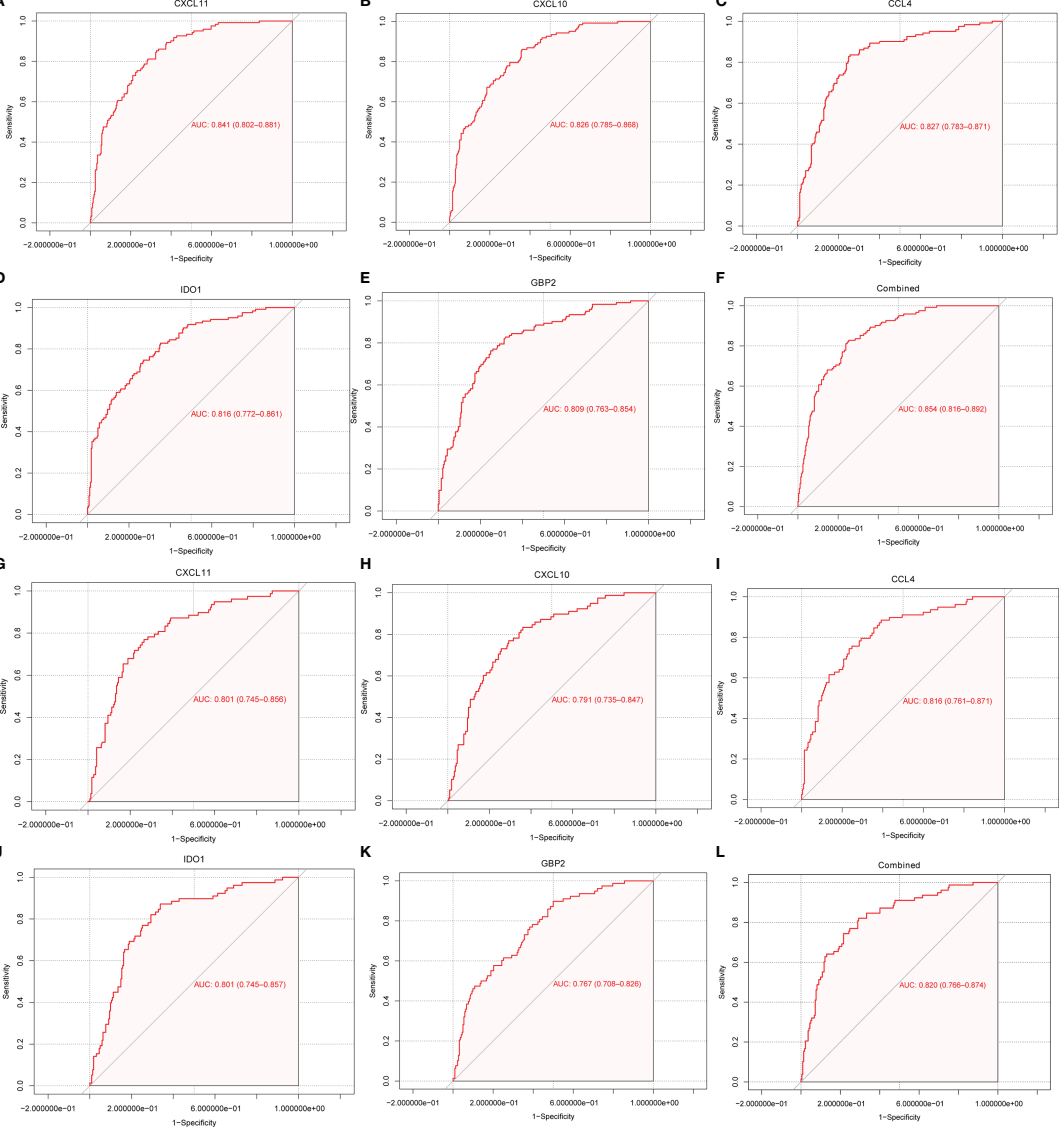
ROC curve for discriminating rejection from no-rejection tissues. **(A–F)** ROC curve of CXCL11, CCL4, CXCL10, IDO1, GBP2 and after fitting to one variable in the discovery cohort. **(G–L)** ROC curve of CXCL11, CCL4, CXCL10, IDO1, GBP2 and after fitting to one variable in the GSE48581 dataset.

To explore the diagnostic values of feature genes in kidney rejection, ROC curve analysis was performed using the R package “pROC”. As shown in **Figures 5A–F**, the AUC was 0.841 (95% CI: 0.802-0.881) for CXCL11, 0.826 (95% CI: 0.785-0.868) for CXCL10, 0.827 (95% CI: 0.783-0.871) for CCL4, 0.816 (95% CI: 0.772-0.861) for IDO1, 0.809 (95% CI: 0.763-0.854) for GBP2, 0.854 (95% CI: 0.816-0.892) when above 5 genes were combined into one variable in the GSE36059 dataset. In the GSE48581 dataset, levels of AUC were also satisfactory (**Figures 5G–L**).

### Correlation Between Gene Expression and Immune Cell Infiltration

**Figure 6:**
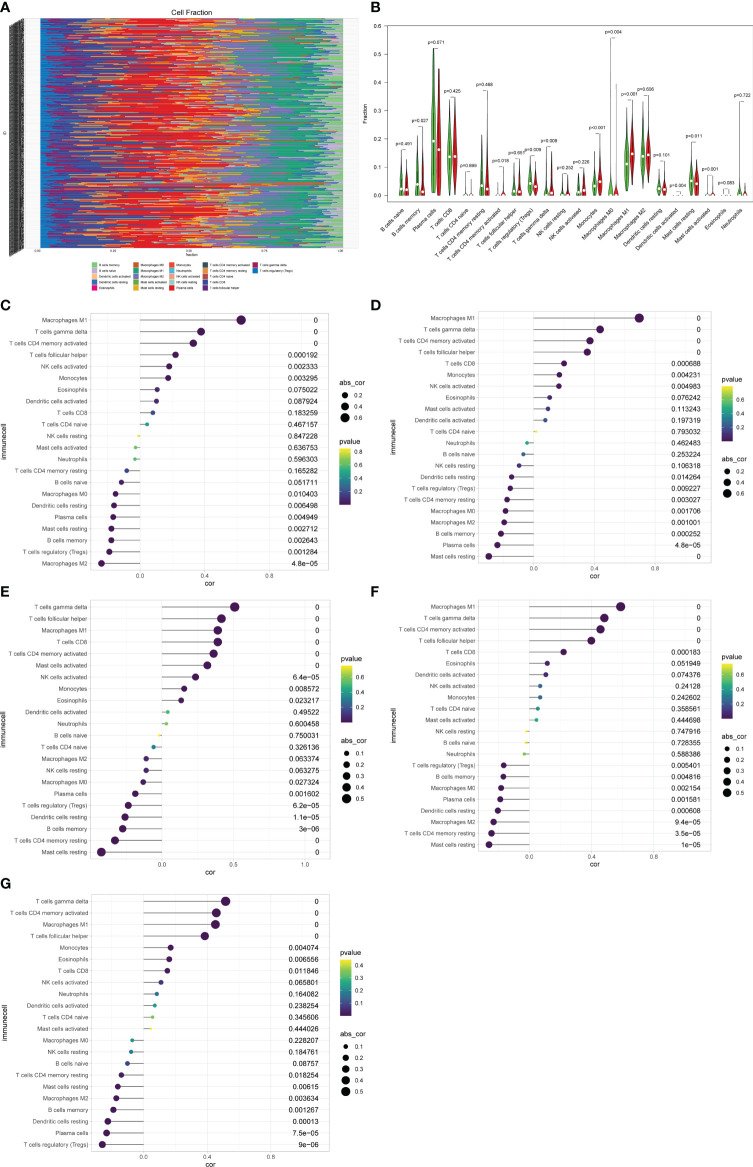
Immune cell proportions in the discovery cohort. **(A)** A bar plot of the immune cell proportions in no-rejection and rejection samples. **(B)** A violin plot of the immune cell proportions in the discovery cohort. Blue and red colors represent no-rejection and rejection samples, respectively. **(C–G)** Correlation between prognostic-related genes and immune cells.

The CIBERSORT algorithm was performed to assess immune cells infiltration in the GSE36059 dataset. 22 subpopulations of immune cells in the GSE36059 dataset are shown in **Figure 6A**. We found that the rejection group had a significantly higher relative percentage of gamma delta T cells, CD4+ activated memory T cells, monocytes, M0 macrophages, M1 macrophages, and activated mast cells in comparison to the non-rejection group (**Figure 6B**). The correlation analysis suggested that CXCL11 had a positive correlation with M1 macrophages (r=0.63, p<0.001), and gamma delta T cells (r=0.37, p<0.001) (**Figure 6C**). CXCL10 had a positive correlation with M1 macrophages (r=0.69, p<0.001), and gamma delta T cells (r=0.43, p<0.001) (**Figure 6D**). CCL4 had a positive correlation with gamma delta T cells (r=0.51, p<0.001), follicular helper T cells (r=0.42, p<0.001), and negative correlation with resting mast cells (r= −0.42, p<0.001) (**Figure 6E**). IDO1 had a positive correlation with M1 macrophages (r=0.59, p<0.001), gamma delta T cells (r=0.48, p<0.001), and CD4+ activated memory T cells (r=0.46, p<0.001) (**Figure 6F**). GBP2 had a positive correlation with gamma delta T cells (r=0.52, p<0.001), CD4+ activated memory T cells (r=0.46, p<0.001), and M1 macrophages (r=0.45, p<0.001) (**Figure 6G**).

### Construction and Validation of an Immune-Related Gene Signature for Graft Loss

**Figure 7:**
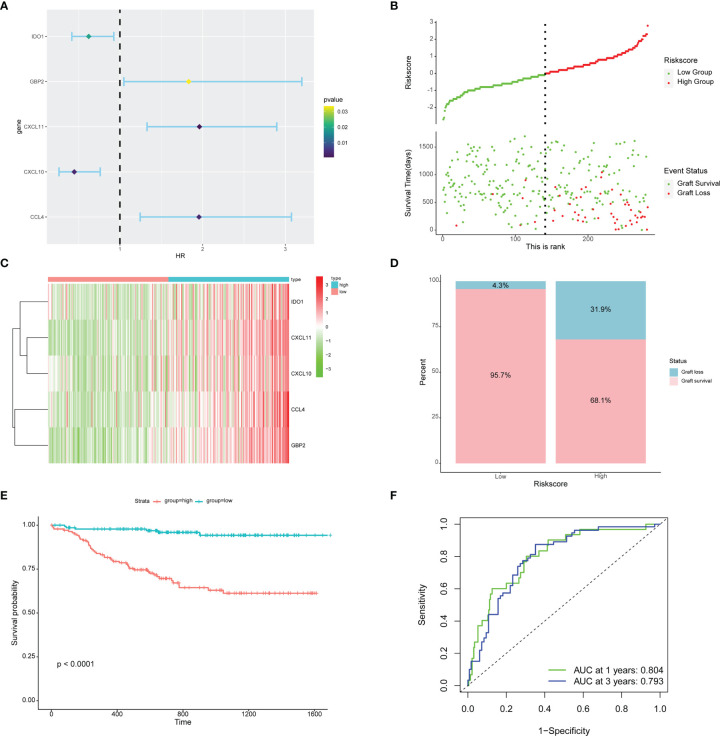
Prognostic risk model building and evaluation. **(A)** Forest plot of five genes related to graft loss analyzed by multivariate Cox regression. **(B)** RiskScore and graft survival time distributions of the low-risk and high-risk groups. **(C)** The heat map of five prognostic-related genes. **(D)** The proportion of recipients stratified by risk groups. **(E)** Survival analysis of recipients in different risk groups. **(F)** The AUC of the ROC.

Based on the SVM-RFE algorithm, 5 genes (CXCL11, CCL4, CXCL10, IDO1, and GBP2) were selected. Univariate Cox regression analysis showed that all 5 genes were related to graft loss (**Supplementary Table 1**). Then, multivariate Cox regression analysis was used to establish an immune-related gene prognostic signature for graft loss (**Figure 7A**). The RiskScore of each recipient was calculated. Patients were divided into the high-risk and low-risk groups according to the median value of RiskScore (**Figure 7B**). All these five genes were upregulated in the high-risk group (**Figure 7C**). The rate of graft failure was higher in the high-risk group compared with the low-risk group (**Figure 7D**). KM survival curves were generated to evaluate the prognostic value of RiskScore. Recipients in the high-risk group had poor graft survival (**Figure 7E**). AUC values of 1- and 3-year graft survival were 0.804 and 0.793 (**Figure 7F**), respectively, indicating that the model had good sensitivity and specificity in predicting the graft loss.

### Correlation Between the RiskScore and Immune Cell Infiltration

**Figure 8:**
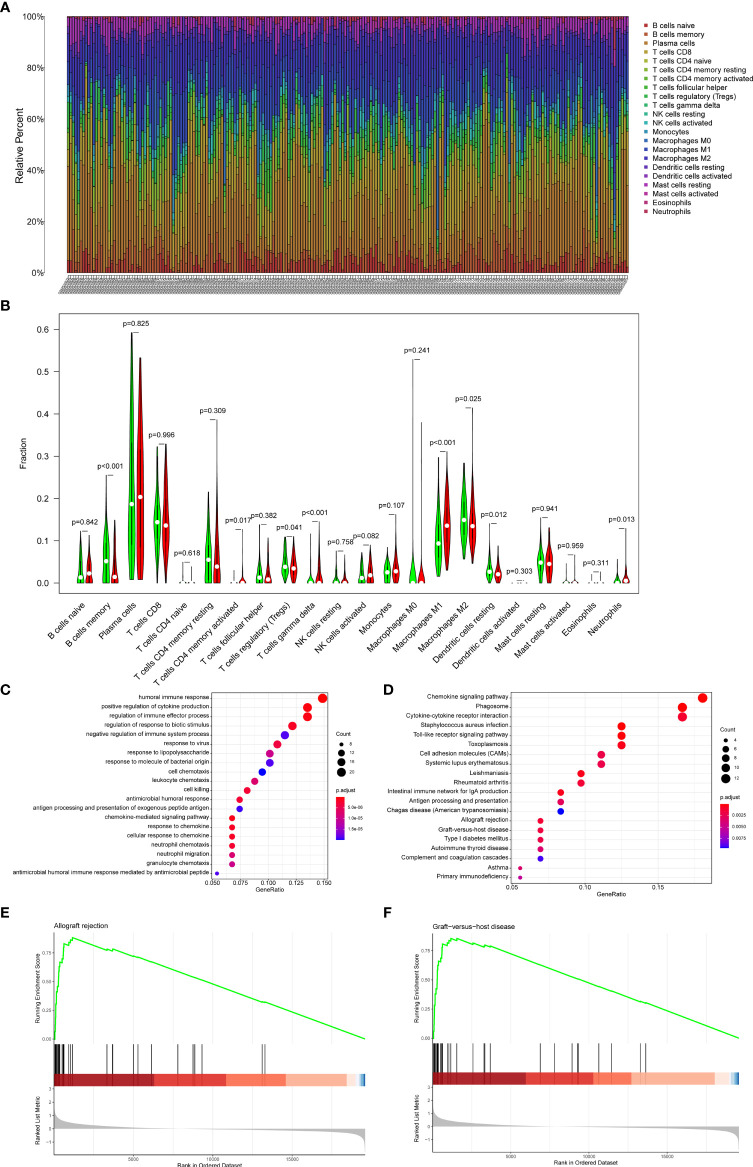
Immune cell proportions in the GSE21374 dataset. **(A)** A bar plot of the immune cell proportions in the low-risk and high-risk groups. **(B)** A violin plot of the immune cell proportions in the low-risk and high-risk groups. Blue and red colors represent low-risk and high-risk samples, respectively. **(C)** GO functional enrichment analyses. **(D)** KEGG functional enrichment analyses. **(E, F)** The GSEA enrichment analysis.

22 subpopulations of immune cells in the GSE21374 dataset are shown in **Figure 8A**. We found that the high-risk group had a significantly higher relative percentage of M1 macrophages, CD4+ activated memory T cells, and gamma delta T cells compared with the low-risk group (**Figure 8B**). The low-risk group had a significantly higher relative percentage of M2 macrophages, resting dendritic cells, Tregs, and memory B cells. The correlation analysis showed that Riskscore had a positive correlation with gamma delta T cells (r=0.42, p<0.001), CD4+ activated memory T cells (r=0.36, p<0.001) (**Supplementary Figure 1**).

### Results of Enrichment Analysis

To explore the potential mechanisms underlying the Riskscore system in recipients, enrichment analyses were performed between the high and low-risk groups. GSEA results showed that allograft rejection and graft-versus-host disease were the most significantly enriched pathways in the high-risk group (**Figures 8E, F**). The GO (**Figure 8C**) and KEGG (**Figure 8D**) analysis indicated that the DEGs between high-risk and low-risk groups were mainly involved in inflammatory pathways, chemokine-related pathways, and rejection-related pathways. These results suggested potential roles for immune-related genes in the process of graft loss.

## Discussion

Graft rejection is a principal risk factor for late graft loss. Immune-related genes play critical roles in graft rejection. We hypothesized that immune-related genes in graft rejection might act as prognostic markers for graft loss. Therefore, some public datasets were used to explore the role of immune-related genes in graft rejection and graft loss after kidney transplantation. To find the immune-related genes that are associated with kidney graft rejection, the GSE36059 dataset was used to detect differentially expressed genes between rejection tissues and non-rejection tissues. Totally 15 genes were obtained. The GO and KEGG analysis showed that these genes were mainly associated with immune-related signal pathways. Then, the SVM-RFE algorithm was performed to select feature genes. After that, CXCL11, CCL4, CXCL10, IDO1, and GBP2 were identified as potential feature genes that were correlated with graft rejection. Finally, a risk model was constructed to predict kidney graft loss based on multivariate Cox regression analyses in the GSE21374 dataset, which showed favorable predictive values in predicting graft loss. In the high-risk group, allograft rejection and graft-versus-host disease were the most significantly enriched pathways. Different immune cell infiltration was also observed between the two groups.

Immune responses against allografts ultimately lead to graft rejection, which has been considered as the main reason for graft loss (4). Immune-related genes play crucial roles in immune cell infiltration and the production of inflammatory cytokines. CXCL11 is a ligand of CXCR3, which is mainly expressed on Th1 effector cells, and is involved in the induction of immune responses against some foreign antigens. CXCR3-chemokine ligand interactions have important roles in inflammatory, autoimmune, feto-maternal immune tolerance, transplant rejection, and graft-versus-host disease (12, 13). CXCL11 is also a main cause of cellular rejection. Its urinary level was found to be increased in both antibody-mediated rejection (ABMR) and T cell-mediated rejection (TCMR) (14, 15). Some previous studies have found that the expression level of CXCL11 was upregulated in rejected skin, lung, and cardiac grafts (12, 13). Our results also confirmed that CXCL11 was upregulated in rejection samples. CXCL10 is a ligand of CXCR3 too, which has been most widely studied and is sensitive to a range of different stimuli (12). Urinary CXCL10 is capable of predicting graft failure or rejection (12). CXCL10 could also act as a biomarker for acute rejection (16, 17). However, there still remains controversial data. In animal models, inhibition of CXCL10/CXCR3 could result in progressive renal fibrosis by upregulating the expression of TGF-β (18). Some investigations have demonstrated that CXCR3-chemokine ligand interaction can be beneficial under certain conditions. It has been previously shown that infiltration of CXCR3 positive regulatory T cells is related to appropriate graft function in kidney allograft (19). Increased CXCR3 ligands could contribute to T cell migration and rejection (20). Consistently, we found that CXCL11 and CXCL10 had a positive correlation with some T cell subtypes. CCL4 is a kind of CXCR5 ligands that is mainly expressed in T and NK cells. It was previously shown that CCL4 could act as an universal rejection marker (21). However, the roles of CCL4 in kidney transplantation need further elaboration due to the low number of studies. IDO1 is a metabolic enzyme, which is related to tryptophan catabolism in plasmacytoid dendritic cells (22, 23). IDO1 is also a universal rejection-associated enzyme (21). IDO1 has long been considered to have immunomodulatory effects (24–27). Treatment with an IDO1 inhibitor was shown to induce graft rejection, and IDO1 may hold promise for suppressing allograft rejection (28). Our results also demonstrated that IDO1 had a protective factor against kidney graft loss. In addition, activation of IDO-expressing DCs was capable of promoting graft survival (29). Several previous studies have demonstrated the protective role of IDO1 in graft-versus-host disease (GVHD) (30, 31). A recent study suggested that myeloid-derived IDO1 had the ability to improve GVHD survival (32). GBP2 was a commonly used gene that distinguished non-rejection and rejection kidney allografts (33). Moreover, it was previously shown that GBP2 could distinguish between rejection and other kinds of liver dysfunction (34). These five genes were associated mainly with innate immune cells, such as M1 macrophages, gamma delta T cells, and monocytes, which highlights the importance of innate immune cells in kidney allograft rejection. Finally, although the majority of studies have suggested that CXCR3 and its ligands were risk factors for graft rejection, controversy in this field still exists. More and better studies are required to better understand the mechanisms of CXCR3 and its ligands in graft rejection and graft failure.

Furthermore, a prognostic model was constructed based on these immune-related genes for predicting graft loss in recipients after kidney transplantation. Time-dependent ROC analysis showed that the Riskscore system had satisfactory performance in the prediction of graft loss. Survival analysis showed significant differences in graft survival between the low-risk and high-risk groups. These results demonstrated the good performance of the model. GSEA showed that allograft rejection and graft-versus-host disease were the most significantly enriched pathways in the high-risk group. GO and KEGG functional enrichment analyses suggested that DEGs between high-risk and low-risk groups were mainly involved in inflammatory pathways, chemokine-related pathways, and rejection-related pathways. These results may partially explain the potential mechanisms underlying the Riskscore system. Moreover, since immune cell infiltration plays an important role in allograft rejection by secreting immune-related cytokines, the CIBERSORT algorithm was applied to assess the level of immune infiltration in the low-risk and high-risk groups. Our results were consistent with previous studies. We observed significant differences in immune cell composition between the two groups. In the high-risk group, we found an increase in the proportion of M1 macrophages, CD4+ activated memory T cells, and gamma delta T cells. In the low-risk group, high levels of M2 macrophages, resting dendritic cells, Tregs, and memory B cells were observed. Previous evidence showed that resting DCs have a regulatory role in protecting against autologous T-cell-mediated autoimmune damage (35). Gamma delta T cells are mediators of the immune response. Some evidence showed that they can be both harmful and beneficial at the same time (36). Their role in kidney transplantation is still unclear. M1 macrophages could produce pro-inflammatory cytokines, which were involved in allograft rejection and result in tissue damage and poor graft survival (37). Our results are in line with this. M2 macrophages play important roles in anti-inflammation in allograft rejection. However, M2 macrophages are reported to contribute to fibrosis, leading to poor long-term graft survival (37). Moreover, macrophages are associated with graft vasculopathy, but the phenotypes remain unidentified (38). Tregs are a subpopulation of CD4+ T cells that have crucial roles in suppressing the immune response, which is essential for the maintenance of immune tolerance (39–41). Depletion of Tregs contributed to the rejection of kidney allografts (42). Our results also showed that the relative level of Tregs was low in the high-risk group compared to the low-risk group. In brief, these five immune-related genes were related to inflammation, graft rejection, immune cell infiltration and may act as prognosis markers for kidney graft loss.

However, some limitations in our study should be noted. First, this study was retrospective, and some clinical information was not available in the GSE21374 dataset. Second, our results were mainly based on GEO database. Therefore, functional experiments are needed to uncover possible regulatory mechanisms of these immune-related genes and complete clinical trials are needed to validate the results of this immune-related prognostic model. Last, the immune cell infiltration in kidney graft tissues was inferred by CIBERSORT, and these predictions need to be confirmed by further studies.

## Conclusions

In conclusion, the current study provided insight into the immune-related genes in kidney graft rejection and an immune-related gene signature was established to predict graft loss after kidney transplantation. Finally, 5 feature genes were identified highly expressed in the rejection group. Based on these genes, an immune-related prognostic model was established. This model had good sensitivity and specificity in predicting the 1- and 3-year kidney graft survival, which might act as a useful tool for predicting kidney graft loss. Analysis of immune infiltrates showed that these five genes were associated mainly with innate immune cells. For further clinical implementation, more evaluation and validation are warranted.

## Data Availability Statement

The datasets presented in this study can be found in online repositories. The names of the repository/repositories and accession number(s) can be found in the article/**Supplementary Material**.

## Author Contributions

MD and PT conceived and designed the study. MD, BZ, GD, KZ, and CD performed the data analysis. MD wrote the original draft. PT, XD, CX, JZ, and WX reviewed and revised the manuscript. All authors contributed to the article and approved the submitted version.

## Funding

This work was supported by the National Nature Science Foundation of China (No. 81870514, No. 81900686, and 82070768).

## Conflict of Interest

The authors declare that the research was conducted in the absence of any commercial or financial relationships that could be construed as a potential conflict of interest.

## Publisher’s Note

All claims expressed in this article are solely those of the authors and do not necessarily represent those of their affiliated organizations, or those of the publisher, the editors and the reviewers. Any product that may be evaluated in this article, or claim that may be made by its manufacturer, is not guaranteed or endorsed by the publisher.
