# Supplementary material for: Immune-Related Genes for Predicting Future Kidney Graft Loss: A Study Based on GEO Database
Source: Front Immunol. 2022 Feb 25;13:859693. doi: 10.3389/fimmu.2022.859693 (PMC8913884; doi:10.3389/fimmu.2022.859693)

Figure S1. Validation of expression level of identified feature genes in the GSE131179 dataset

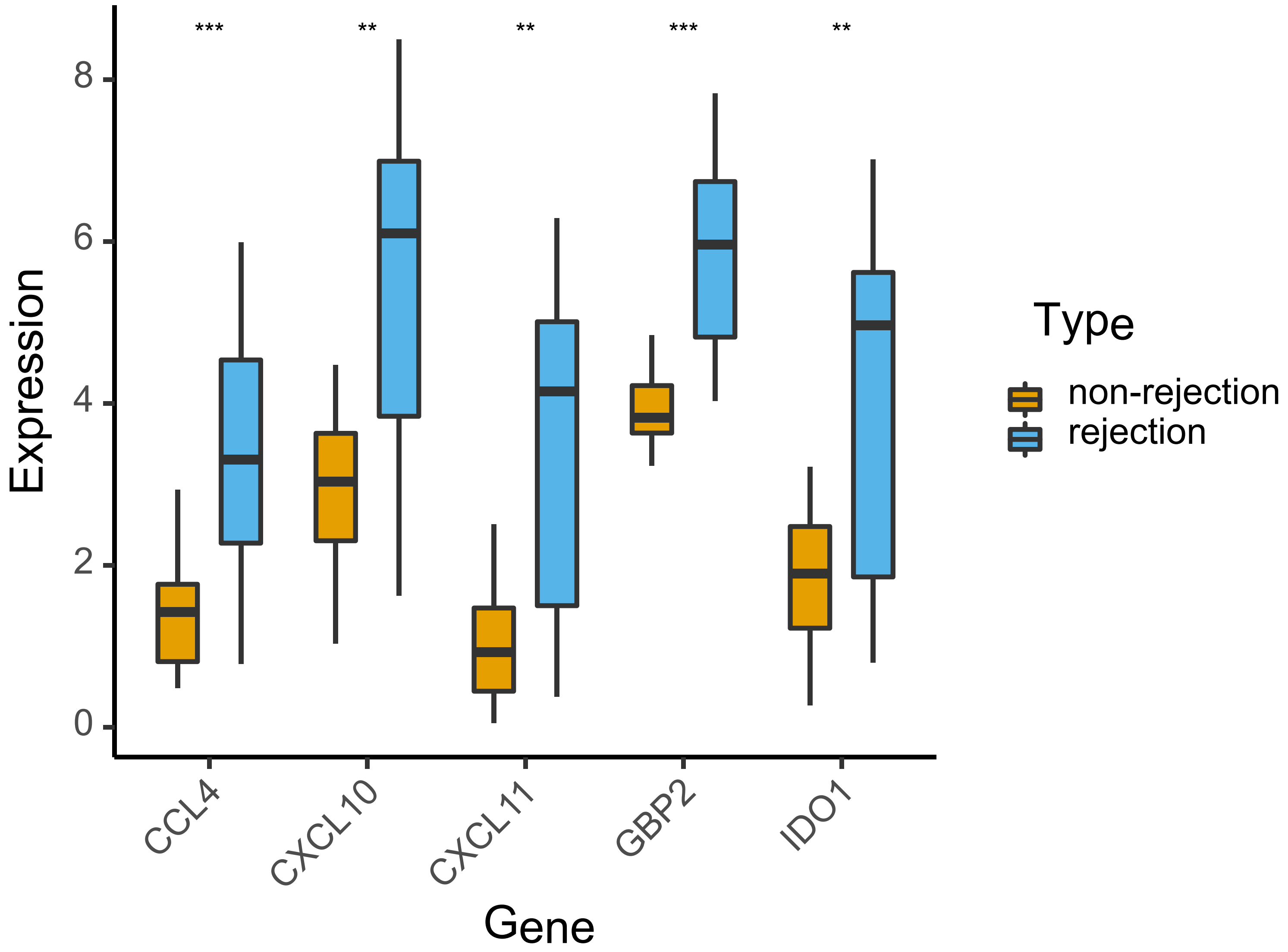

Figure S2. Validation of expression level of identified feature genes in the GSE50058 dataset

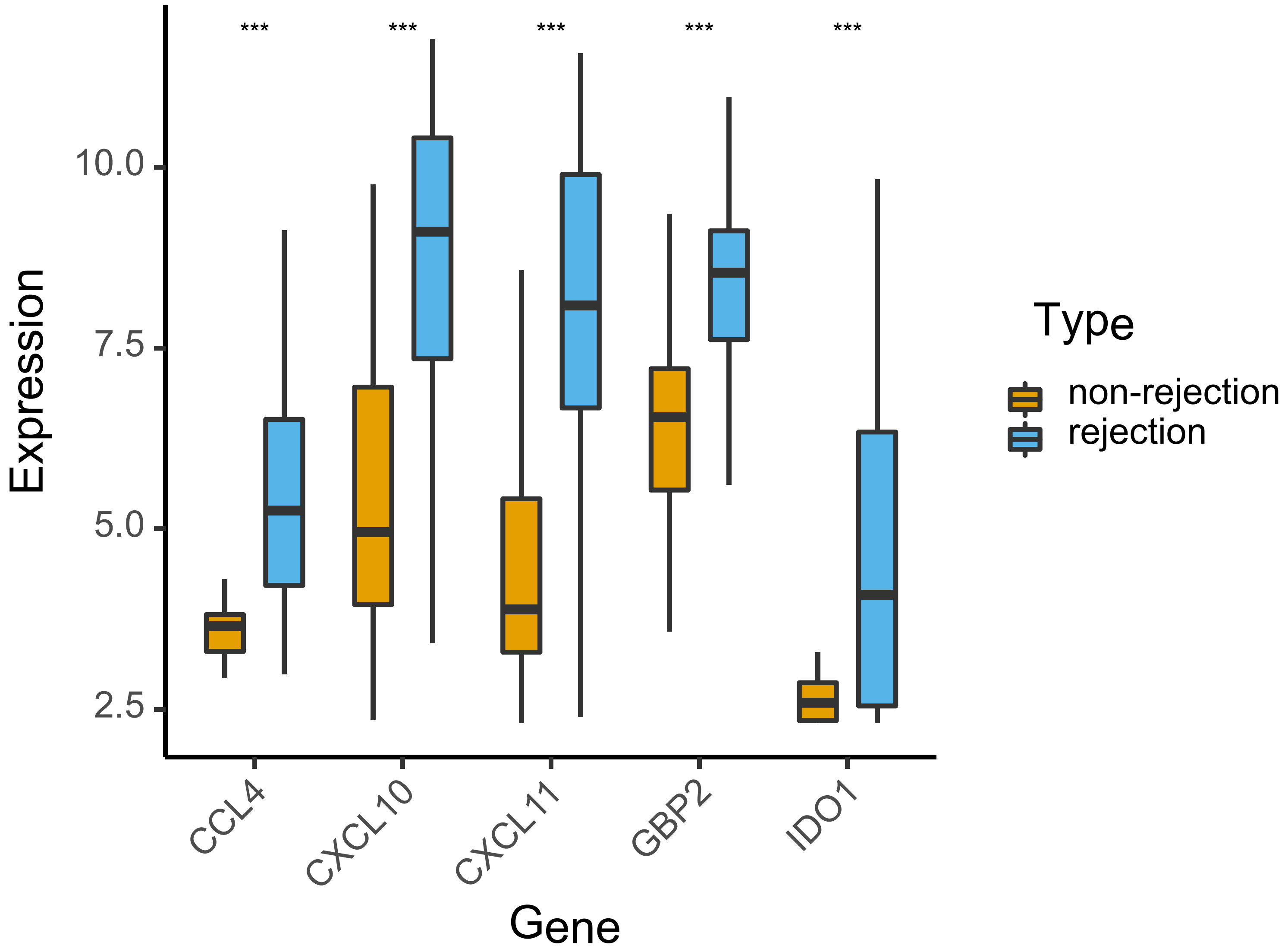

Figure S3. Validation of expression level of identified feature genes in the GSE72925 dataset

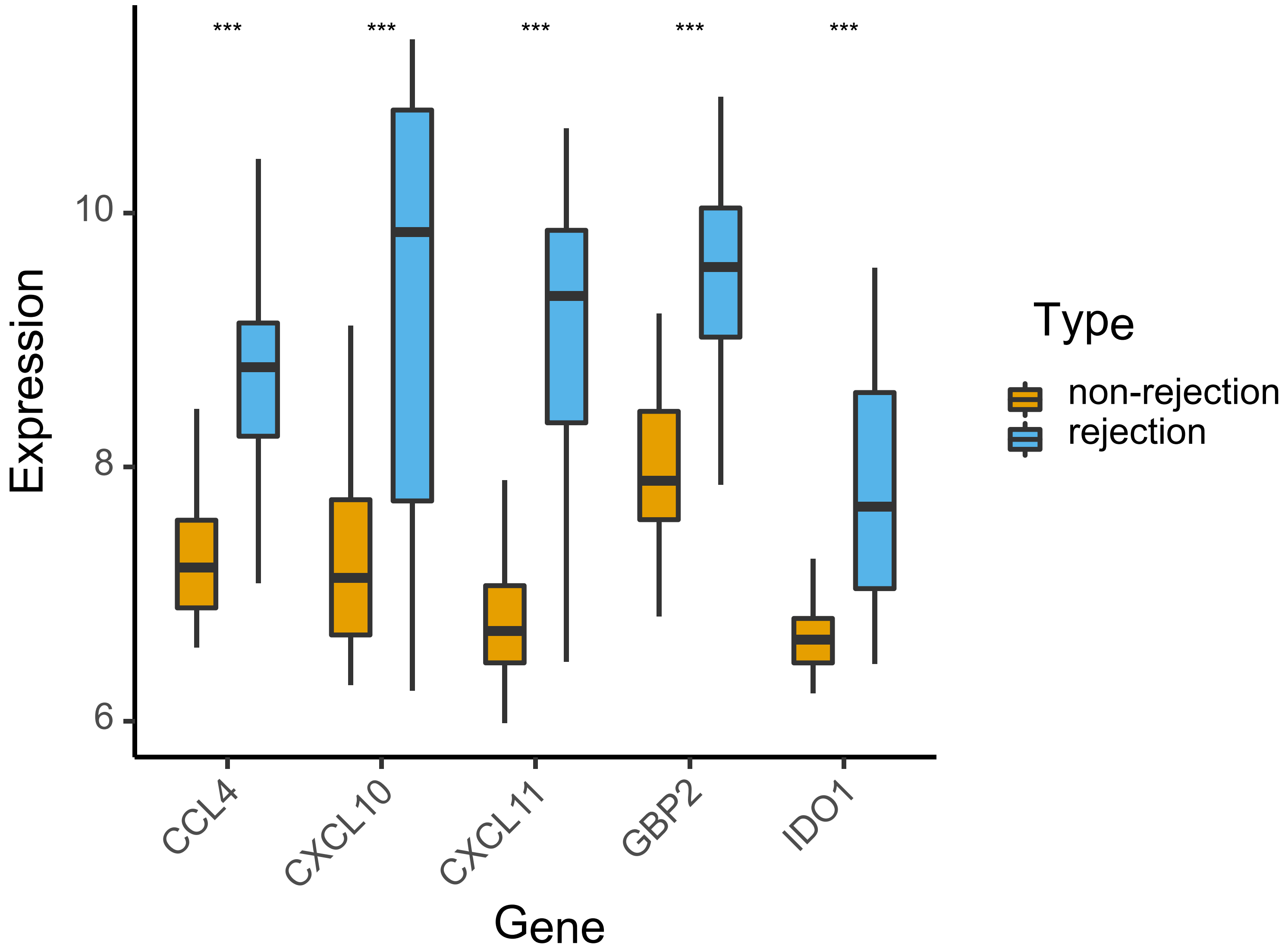

Figure S4. Validation of expression level of identified feature genes in the GSE75693 dataset

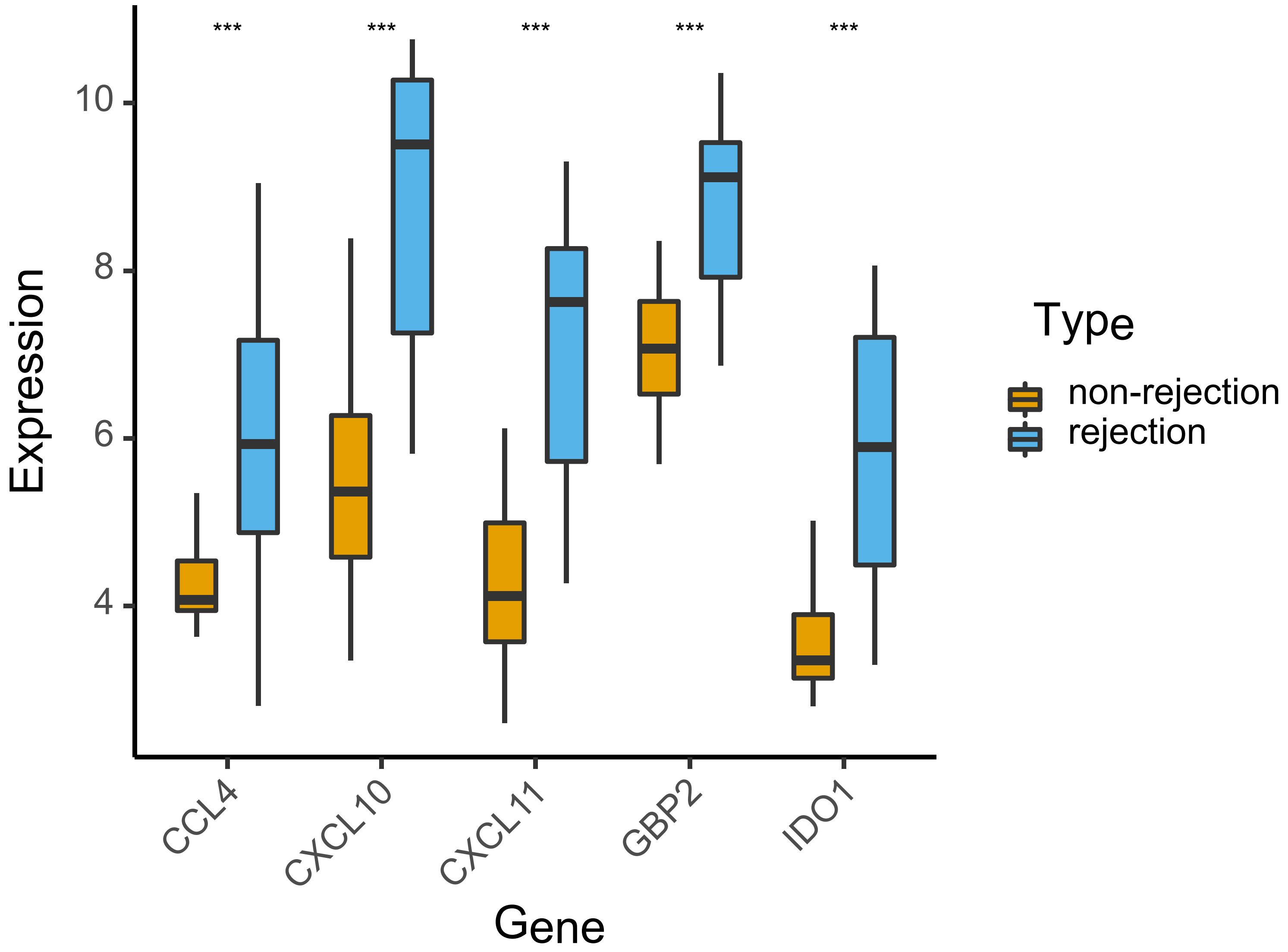

Figure S5. Validation of expression level of identified feature genes in the GSE9493 dataset

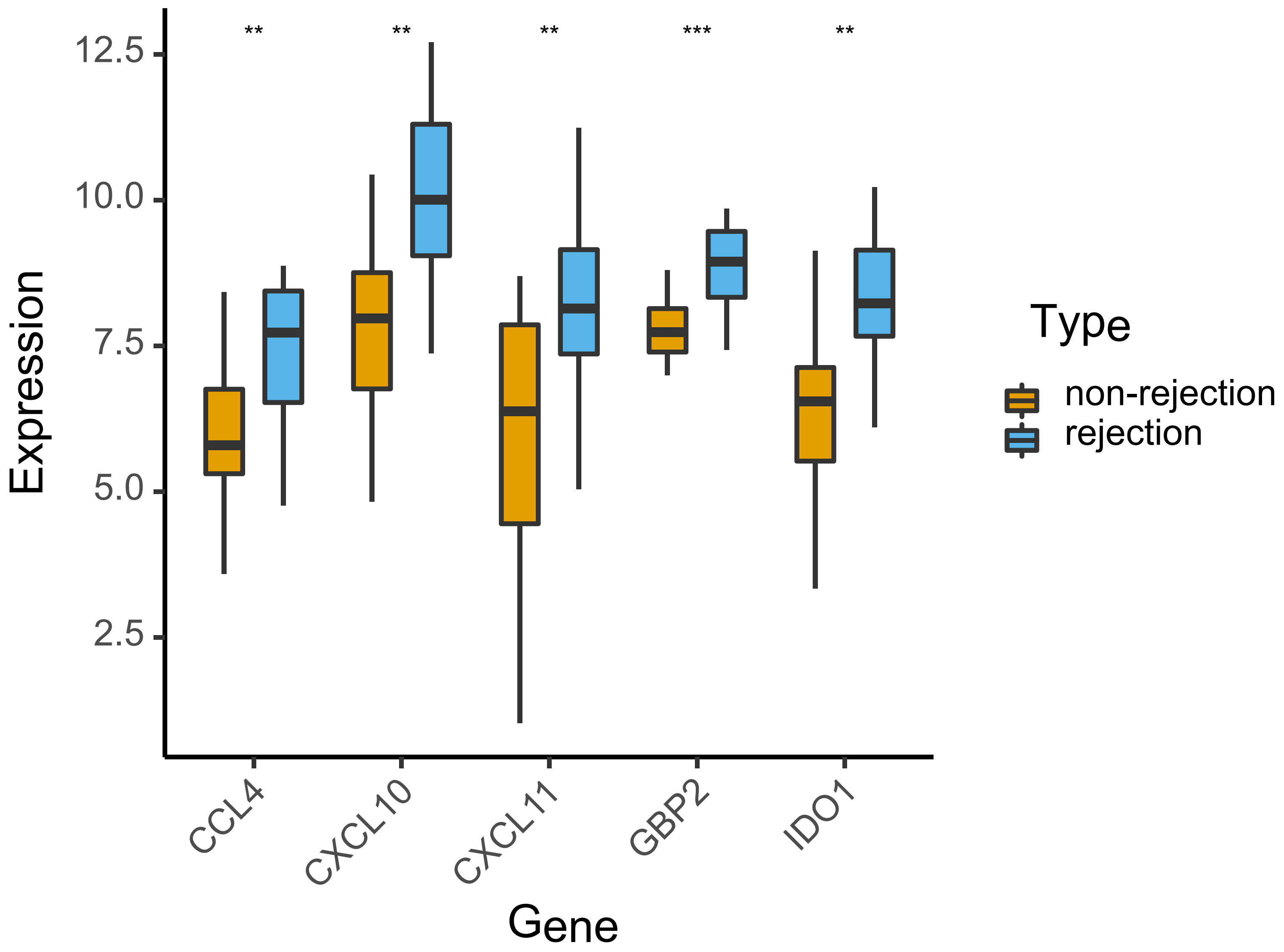

Supplement: Supplementary file 1 [file DataSheet_1.zip › Supplementary material/File S1.pdf]
