# Supplementary material for: Immune-Related Genes for Predicting Future Kidney Graft Loss: A Study Based on GEO Database
Source: Front Immunol. 2022 Feb 25;13:859693. doi: 10.3389/fimmu.2022.859693 (PMC8913884; doi:10.3389/fimmu.2022.859693)

Figure S6. CXCL11 in sing-cell level

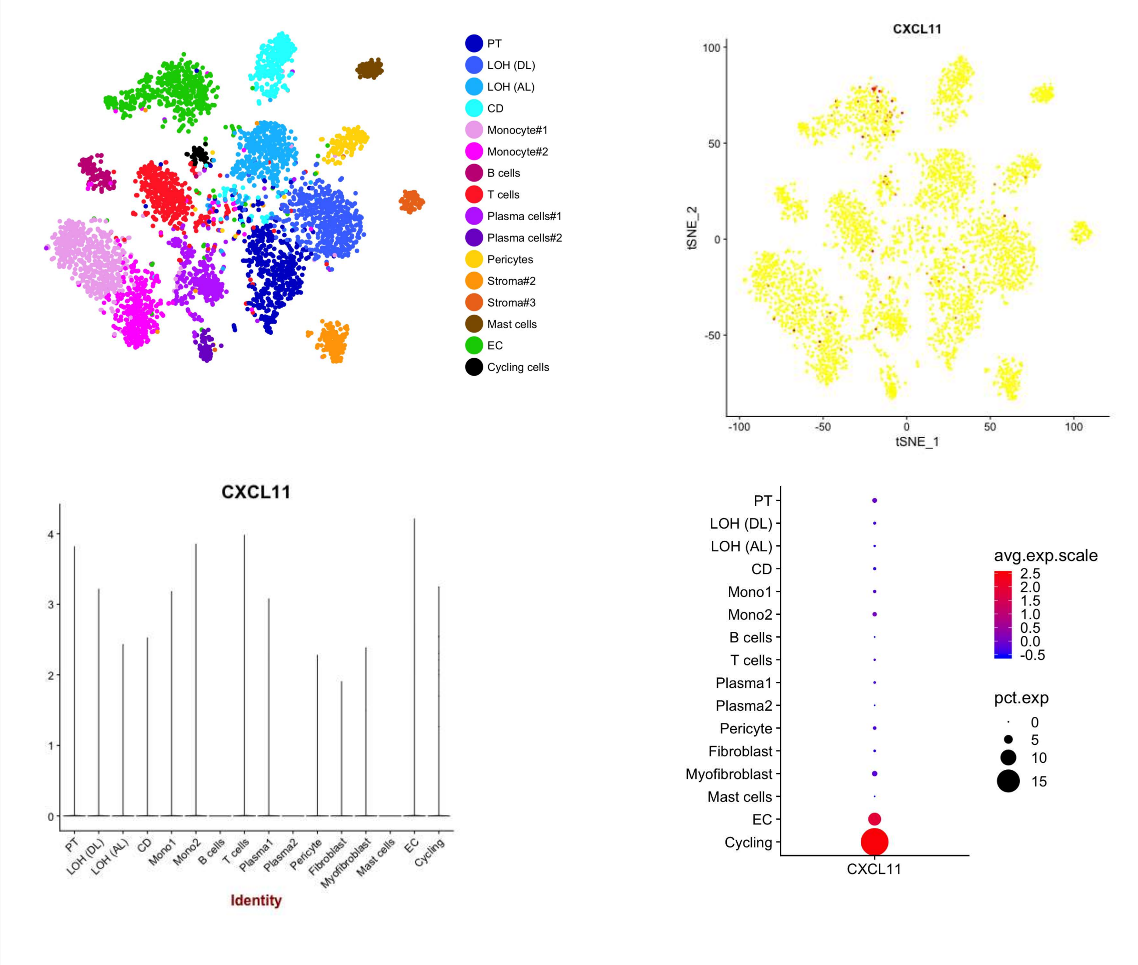

Figure S7. CXCL10 in sing-cell level

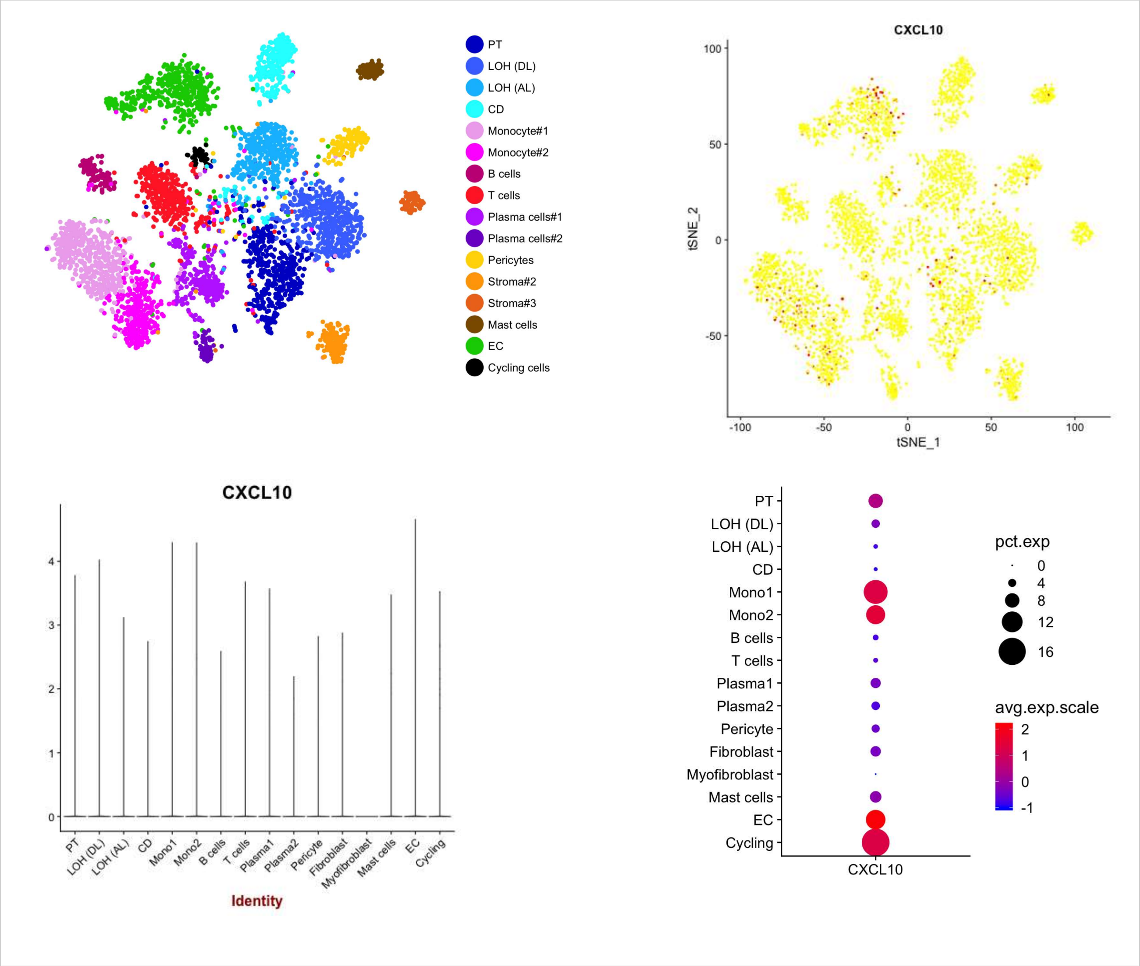

Figure S8. CCL4 in sing-cell level

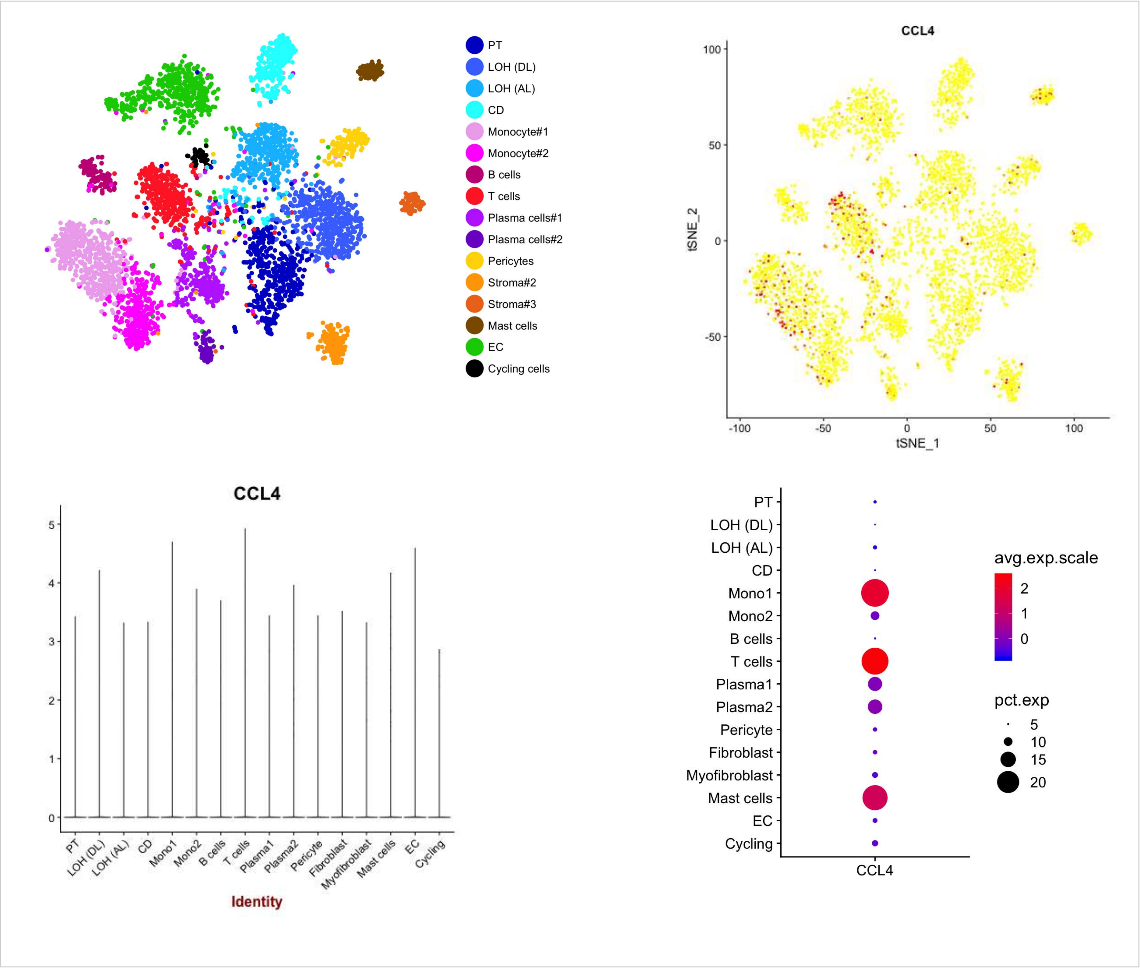

Figure S9. IDO1 in sing-cell level

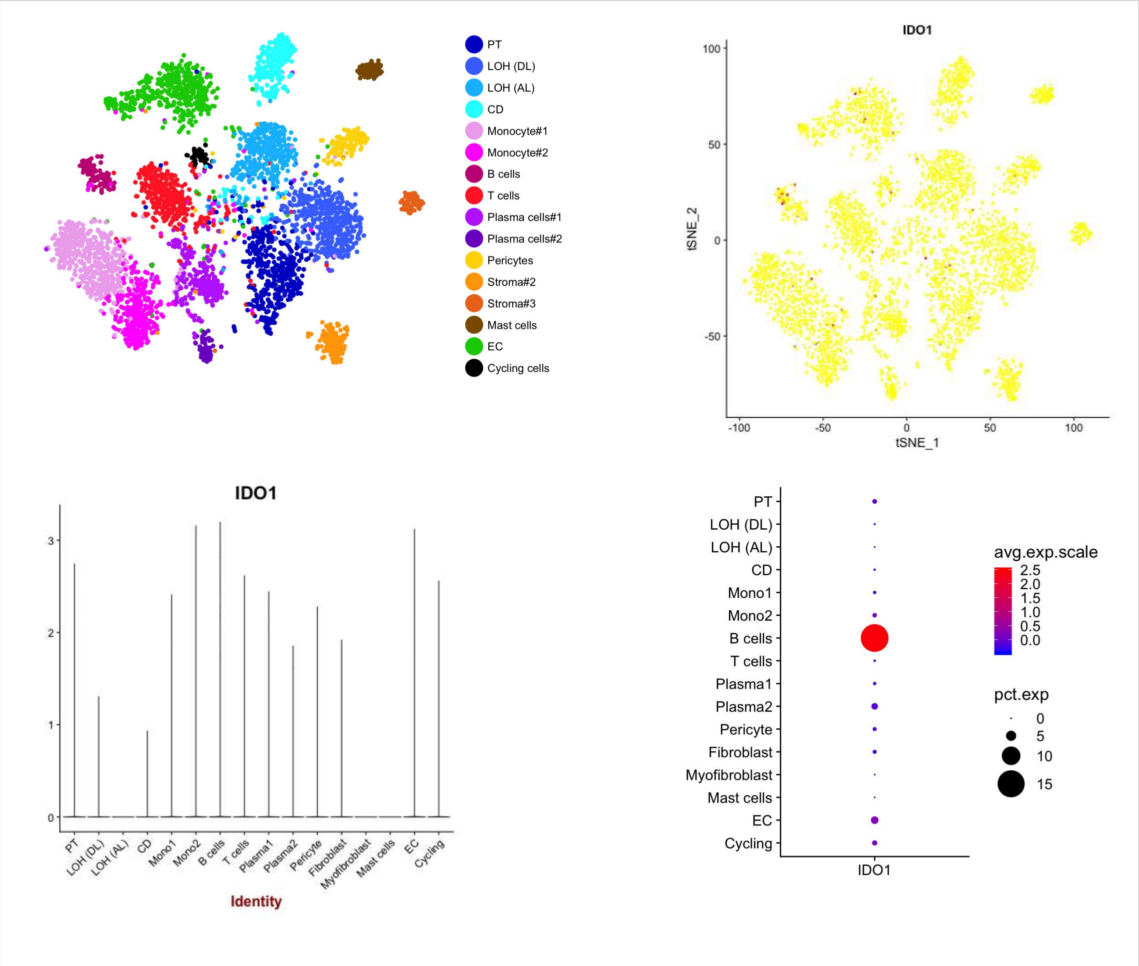

Figure S10. GBP2 in sing-cell level

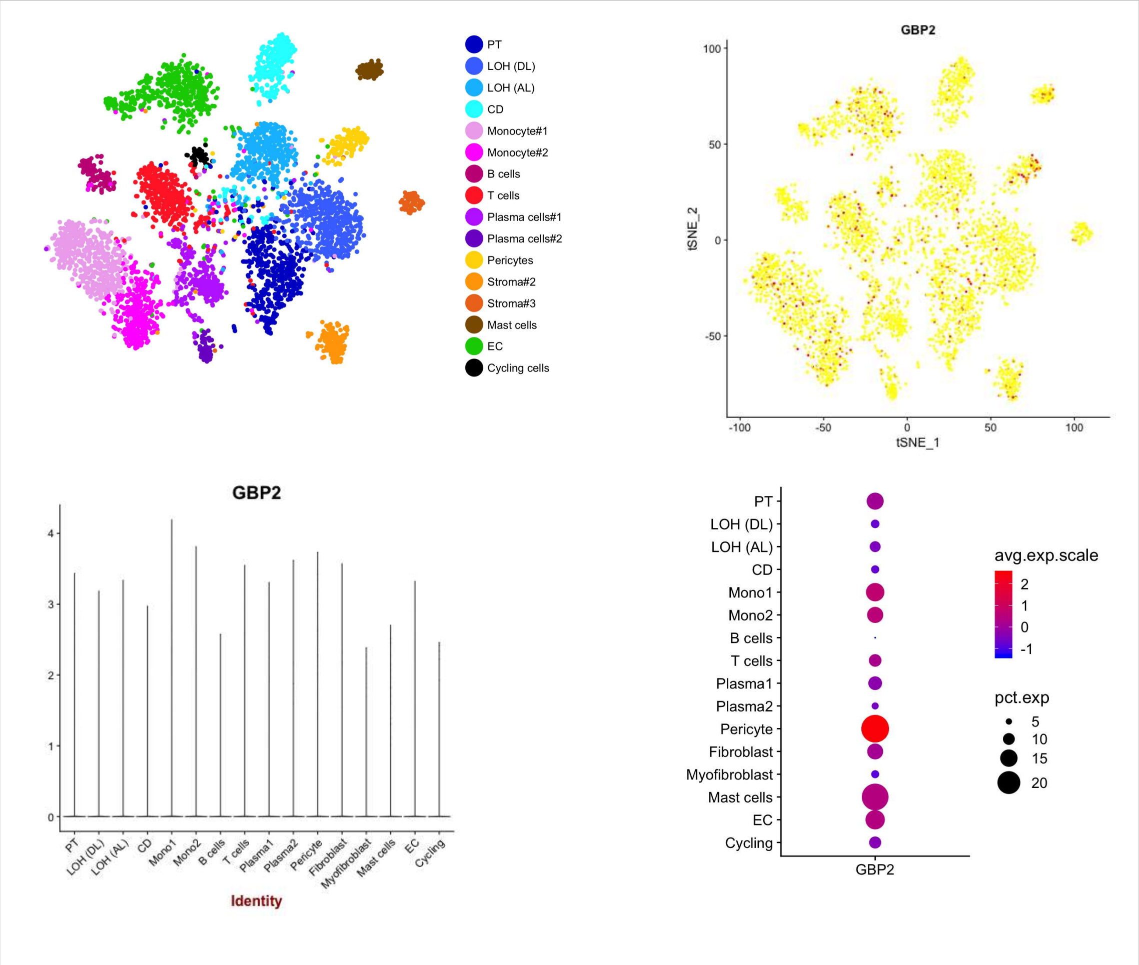

Supplement: Supplementary file 1 [file DataSheet_1.zip › Supplementary material/File S2.pdf]
