# Supplementary material for: Immune-Related Genes for Predicting Future Kidney Graft Loss: A Study Based on GEO Database
Source: Front Immunol. 2022 Feb 25;13:859693. doi: 10.3389/fimmu.2022.859693 (PMC8913884; doi:10.3389/fimmu.2022.859693)
Supplement: Supplementary file 1 [file DataSheet_1.zip › Supplementary material/File S3 R code.pdf]

1. discovery cohort

```
library(tidyverse)
library(pacman)
library(openxlsx)
library(toudouStringr)
library("docxR")
library(data.table)
library(future.apply)
library(clipr)
library(ggpubr)
library(DataExplorer)
library(psych)
library(eoffice)
library(RColorBrewer)
library(data.table)
library(limma)

# DE-IRGs in the discovery cohort ----------------------------------------

getDEG <- function(gset,before,after,GSE){
  group_list=c(rep('before',before),rep('after',after))
  group_list=factor(group_list)
  group_list=relevel(group_list,ref = "before")
  boxplot(gset,outline=FALSE, notch=T,las=2,col=group_list)
  library(limma)
  design=model.matrix(~ group_list)
  fit=lmFit(gset,design)
```

```r
  fit=eBayes(fit)
  allDiff=topTable(fit,adjust='fdr',coef="group_listafter",number=Inf,p.value=)
  library(openxlsx)
  write.xlsx(allDiff,file =paste(GSE,"DE-IRGs1.xlsx",sep="_") ,rowNames = TRUE)
  gset=normalizeBetweenArrays(gset)
  boxplot(gset,outline=FALSE, notch=T,las=2,col=group_list)
  design=model.matrix(~ group_list)
  fit=lmFit(gset,design)
  fit=eBayes(fit)
  allDiff_jiaozheng=topTable(fit,adjust='fdr',coef="group_listafter",number=Inf,p.value=)
  write.xlsx(allDiff_jiaozheng,file = paste(GSE,"DE-IRGs1.xlsx",sep="_"),rowNames =
TRUE)
  library(beepr)
  beep(8)
}

gset <- gset_mianyi[,read.delim("clipboard")$ID]

getDEG(gset,281,122,"DE-IRGs_discovery")



# SVM-RFE Analysis -------------------------------------------------------

rm(list = ls())
library(caret)
library(tidyverse)
library(clipr)
my_gene <- read.delim("clipboard")[,1]

my_juzheng <- my_clinical_mianyi2 %>% dplyr::select(group,my_gene)
```

```
rt <- my_juzheng
control <- rfeControl(functions = caretFuncs, method = "cv", number = 5)
str(rt)
library(DataExplorer)
plot_missing(rt)

set.seed(156)
results <- rfe(rt[,-1],
               rt[,1],
               sizes = c(1:8),
               rfeControl = control,
               method = "svmRadial")
beepr::beep(8)

print(results)

predictors(results)

plot(results, type=c("g", "o"),col="red")
abm <- predictors(results)
write_clip(abm)

# Volcano plots    -------------------------------------------------------

library(ggplot2)
library(ggrepel)
library(tidyverse)
my_gene <- read.delim("clipboard")
de_result <- my_gene
```

```
names(de_result)
de_result <- de_result %>% rename(id=gene,
                                  log2FoldChange=logFC,
                                  pvalue=P.Value,
                                  padj=adj.P.Val)


de_result <- de_result %>% mutate(
  direction=case_when(
    padj<0.05&log2FoldChange>1~"up",
    padj<0.05&-1>log2FoldChange~"down",
    TRUE~"ns"
  )
)
table(de_result$direction)

top_de <- filter(de_result,
                 abs(log2FoldChange) > 1 & padj < 0.05)


my_palette <- c('#4DBBD5FF','#999999','#E64B35FF')
my_palette <- c('#4DBBD5FF','#E64B35FF')
ggplot(data = de_result,aes(x = log2FoldChange ,y = -log10(padj)))+
  geom_point(aes(color = direction,
                 size = abs(log2FoldChange))) +
  geom_hline(yintercept = -log10(0.05),linetype = 'dashed') +
  #geom_label_repel(data = top_de,aes(label = id),label.size = 0.05) +
  geom_vline(xintercept = c(-1,1),linetype = 'dashed') +
  scale_color_manual(values = my_palette) +
  scale_size(range = c(0.1,3)) +
```

```
  labs(x = 'log2 fold change',
         y = '-log10(pvalue)',
         title = 'Vocano plot',
         size = 'log2 fold change') +
  ylim(c(0,40)) +
  guides(size = "none") +
  theme_bw() +
  theme(plot.title = element_text(size = 18,hjust = 0.5),
          legend.background =    element_blank(),
          legend.key = element_blank() ,
          legend.position = c(0.93,0.9))



eoffice::topptx(filename = "Volcano.pptx")


### Volcano2
library(tinyarray)


head(my_gene) #


draw_volcano2(my_gene,
              pkg = 4,#1,2,3,4 respectively means
"DESeq2","edgeR","limma(voom)","limma"
              pvalue_cutoff = 0.05,
              logFC_cutoff = 1,
              adjust = TRUE,
              symmetry = T,
              color = c("blue", "red", "red")
)+labs(x = 'log2 fold change',
         y = '-log10(pvalue)',
```

```
        title = 'Vocano plot',
        size = 'log2 fold change')




draw_volcano(my_gene,
            pkg = 4,#1,2,3,4 respectively means "DESeq2","edgeR","limma(voom)","limma"
            pvalue_cutoff = 0.05,
            logFC_cutoff = 1,
            #lab = NA,
            adjust = F,
            symmetry = T,
            color = c("darkgreen", "darkgrey", "#B2182B"),
)

eoffice::topptx(filename = "Volcano2.pptx")


###Volcano3

head(my_gene)

data <- my_gene%>%select(logFC,adj.P.Val)
logFC_cutoff <- 1
adj.p_cutoff <- 0.05

data$Threshold=as.factor(ifelse(data$adj.P.Val< adj.p_cutoff & abs(data$logFC)>=logFC_cutoff,
```

```
                                    ifelse(data$logFC>
logFC_cutoff,'Up','Down'),'None'))

range(data$logFC)
ggplot(data = data,aes(x = logFC,y=-log10(adj.P.Val),colour=Threshold,size=abs(logFC))) +
  geom_point(alpha=0.4) +
  geom_vline(xintercept=c(-logFC_cutoff,logFC_cutoff),lty=2,col="black",lwd=0.4) +
  geom_hline(yintercept = -log10(adj.p_cutoff),lty=2,col="black",lwd=0.4)+
  scale_color_manual(values=c("blue", "red","red"))+
  theme_bw()+



  theme(axis.title = element_text(size = 15),
        axis.text = element_text(size = 12),
        legend.title = element_text(size = 15),
        legend.text = element_text(size = 12),
        legend.position =c(1,1) ,
        legend.justification = c(1, 1),
        legend.background = element_rect(fill = NULL, colour = "black"))+
  xlim(-4,4)+
  guides(size=F)

eoffice::topptx(filename = "Volcano3.pptx",width=8)



# boxplot ---------------------------------------------------------------



names(my_clinical_mianyi2)
```

```
my_exp_cli_15 <- my_clinical_mianyi2 %>% select(group,mianyi_gene)
my_seed156 <- read.delim("clipboard")[,1]
my_seed156_exp_cli <- my_exp_cli_15 %>% select(group,my_seed156)

###

data <- gather(my_seed156_exp_cli,key = "key",value = "value",-group)
colnames(data)=c("Type","Gene","Expression")

p=ggboxplot(data, x="Gene", y="Expression", color = "Type",
            ylab="Gene expression",
            xlab="",
            legend.title="Type",
            #fill = "Type",
            palette = c("blue","red"),
            width=0.6, add = "none")

p=p+rotate_x_text(60)
p1=p+stat_compare_means(aes(group=Type),
                        method="wilcox.test",
                        symnum.args=list(cutpoints = c(0, 0.001, 0.01, 0.05, 1),
symbols = c("***", "**", "*", " ")),
                        label = "p.signif")

p1
eoffice::topptx(filename = "boxplot_1.pptx",width = 8)
##
library(grafify)

p_3d <- plot_3d_scatterbox(data = data,
```

```
                    xcol = Gene,
                    ycol = Expression,
                    shapes = Type,
                    symsize = 0,
                    symthick=0,
                    jitter = 0,
                    TextXAngle = 45)
p_3d+stat_compare_means(aes(group=Type),
                  method="wilcox.test",
                  symnum.args=list(cutpoints = c(0, 0.001, 0.01, 0.05, 1),
symbols = c("***", "**", "*", " ")),
                  label = "p.signif")

eoffice::topptx(filename = "boxplot_2.pptx",width = 8)



# heat map ---------------------------------------------------------------

library(pheatmap)

inputfile=my_danduo_exp_cli

rt=inputfile
rt <- as.data.frame(rt)
library(tidyverse)
rt <- rt %>%arrange(group)
rt1=rt[-1]
rt1=t(rt1)
annotation=rt[1]
pdf(file=heatmapFile,width = 7,height = 4)
```

```
pheatmap::pheatmap(rt1,
                   annotation=annotation,
                   cluster_cols = FALSE,
                   fontsize_row=8,
                   show_colnames = F,
                   fontsize_col=3,
                   fontsize=7,
                   scale="row",
                   color = colorRampPalette(c("green", "white", "red"))(50) )
dev.off()


get_retu <- function(inputfile,filename=NULL){
  rt=inputfile
  rt <- as.data.frame(rt)
  library(tidyverse)
  rt <- rt %>%arrange(group)
  rt1=rt[-1]
  rt1=t(rt1)
  annotation=rt[1]
  name <- paste(filename,".pdf",sep = "")
  pdf(file=name,width = 7,height = 4)
  pheatmap::pheatmap(rt1,
                     annotation=annotation,
                     cluster_cols = FALSE,
                     fontsize_row=8,
                     show_colnames = F,
                     fontsize_col=3,
                     fontsize=7,
                     scale="row",
```

```
                                   color = colorRampPalette(c("green", "white", "red"))(50) )
  dev.off()

}

get_retu(my_seed156_exp_cli,filename = "heatmap")



# ROC ----------------------------------------------------------------

#

my_seed156_exp_cli$group
my_seed156_exp_cli$group <- ifelse(my_seed156_exp_cli$group=="non-rejection",0,1)
table(my_seed156_exp_cli$group)
names(my_seed156_exp_cli)[1] <- "fenzu"
##
library(pROC)
out <- data.frame()
mydata2 <- my_seed156_exp_cli
for(i in 2:6) {
  data <- mydata2
  ROC1<- roc(data$fenzu,data[,i])
  cell <- names(data)[i]
  auc <- auc(ROC1)
  ciauclow <- ci(auc(ROC1))[1]
  ciauchigh <- ci(auc(ROC1))[3]
  out <- rbind(out,cbind(cell,auc,ciauclow,ciauchigh))
}
```

```
save(out,file = "out.Rdata")

write_clip(out)



abd <- 2:6

out <- list()

for(i in abd) {

  library(pROC)

  data <- mydata2

  ROC1<- roc(data$fenzu,data[,i],smooth=FALSE,ci=T,auc = T)

  file_name1 <- paste(names(data)[i],"svm.pdf",sep = "_")

  # file_name1 <- paste(dir,file_name1,sep = "")

  pdf(file=file_name1, width=8, height=6)

  plot(ROC1,

         add = FALSE,

         col = "red",

         legacy.axes = TRUE,

         xlab = "1-Specificity",

         main = names(data)[i],

         print.auc =TRUE,

         auc.polygon = TRUE,

         auc.polygon.col = "#fff7f7",

         grid = c(0.5, 0.2),

         grid.col = c("black", "black"),

         print.thres = FALSE)

  dev.off()

}


names(mydata2)[abd]
```

```
### Logistic
GetFactors <- names(mydata2)[abd]

fml <- as.formula(paste('fenzu',
                        '~',
                        paste(GetFactors,collapse = '+'),collapse = ' '))

fit2 <- glm(fml,x=T,y=T,data = mydata2, family = "binomial")

library("pROC") ##  加载包
aSAH <- mydata2
roc1 <- roc(aSAH$fenzu, fit2$fitted.values, smooth=FALSE,ci=T,auc = T)
plot(roc1,
     add = FALSE,
     col = "red",
     legacy.axes = TRUE,
     xlab = "1-Specificity",
     main = "Combined",
     print.auc =TRUE,
     auc.polygon = TRUE,
     auc.polygon.col = "#fff7f7",
     grid = c(0.5, 0.2),
     grid.col = c("black", "black"),
     print.thres = FALSE)
```

2. GSE21374 dataset

```
library(tidyverse)
library(pacman)
library(openxlsx)
library(toudouStringr)
library("docxR")
library(data.table)
library(future.apply)
library(clipr)
library(ggpubr)
library(DataExplorer)
library(psych)
library(eoffice)
library(RColorBrewer)
library(data.table)
library(limma)


# Forest -----------------------------------------------------------------


#install.packages("ggstatsplot")
library(ggstatsplot)
library(viridis)
HR <- feng_fuben$HR
CI_LL    <-feng_fuben$HR.95L
CI_HL <-feng_fuben$HR.95H
names(feng_fuben)
```

```
ggplot(data=feng_fuben,
            aes(x=HR,y=gene,
                  color=pvalue))+
   geom_errorbarh(aes(xmax=CI_HL, xmin=CI_LL),
                           color="skyblue",height=0.2,size=1.2)+
   geom_point(aes(x=HR,y=gene),size=4,shape=18)+
   geom_vline(xintercept = 1,linetype='dashed',size=1.2, color = "black")+
   scale_color_viridis()

topptx(filename = "Forest_plot.pptx",width=8)




# riskscore -----------------------------------------------------------

library(survival)

data <-    rt
names(data)[1:2] <- get_string(status,time)


cox <- coxph(Surv(time, status) ~CXCL11+CCL4+CXCL10+IDO1+GBP2, data)
coxsummary <- summary(cox)
genes <-    rownames(coxsummary$coefficients)
genes
riskscore <-    predict(cox, type = "risk", newdata = data)
risk<- ifelse(riskscore>median(riskscore),"high","low")
riskresult <-    cbind(patient = rownames(data),data[,c("time","status",genes)], riskscore, risk)
```

```
riskresult_seed156 <- riskresult




rm(list = ls())
names(riskresult_seed156)
rt <- riskresult_seed156 %>% select(-c("patient","risk")) %>%
  rename(
    futime=time,
    fustat=status
  )
res2 <- data.frame()

genes <- colnames(rt)[-c(1:2)]

for (i in 1:length(genes)) {
  print(i)
  group = ifelse(rt[,genes[i]]>median(rt[,genes[i]]),"high","low")
  if(length(table(group))==1) next
  surv =as.formula(paste('Surv(futime, fustat)~', "group"))
  data = cbind(rt[,1:2],group)

  x = survdiff(surv, data = data)

  pValue=1-pchisq(x$chisq,df=1)

  res2[i,1] = genes[i]

  res2[i,2] = pValue
```

```
}
names(res2) <- c("ID","pValue_log")
write_clip(res2)



library(dplyr)
res5 <- res2 %>%
  filter(pValue_log < 0.05)%>%
  arrange(pValue_log)


index <- res5$ID[1]
group = ifelse(rt[,index]>median(rt[,index]),"high","low")
surv =as.formula(paste('Surv(futime, fustat)~', "group"))
data = cbind(rt[,1:2],group)
my.surv <- Surv(rt$futime, rt$fustat)
fit <- survfit(my.surv ~ group)
library(survminer)
ggsurvplot(fit, data = data,pval = TRUE)

topptx(filename = "riskscore.pptx",width = 8)



# ggrisk ----------------------------------------------------------------

names(riskresult_seed156)
library(ggrisk)
library(survival)
library(rms)
```

```
str(riskresult_seed156)
riskresult_seed156 <- riskresult_seed156 %>% dplyr::select(-c("patient","riskscore", "risk"))


fit1                                                                                    <-
rms::cph(Surv(time,status)~CXCL11+CCL4+CXCL10+GBP2+IDO1,riskresult_seed156)
class(fit1)
ggrisk(fit1)
predict(fit1, LIRI[1,c('ANLN', 'CENPA', 'GPR182', 'BCO2')])

fit2                                                                                    <-
rms::cph(Surv(time,status)~CXCL11+CCL4+CXCL10+GBP2+IDO1,riskresult_seed156)
ggrisk(fit2,
       cutoff.value='median',
       cutoff.x = 145,
       cutoff.y = -0.8,
       code.0 = 'Still Alive',
       code.1 = 'Already Dead',
       code.highrisk = 'High Risk',
       code.lowrisk = 'Low Risk',
       title.A.ylab='Risk Score',
       title.B.ylab='Survival Time(year)',
       title.A.legend='Risk Group',
       title.B.legend='Status',
       title.C.legend='Expression')

ggrisk(fit2,
       cutoff.value='median',
       cutoff.x = 145,
       cutoff.y = -0.8,
```

```
code.0 = 'Still Alive',
code.1 = 'Already Dead',
code.highrisk = 'High Risk',
code.lowrisk = 'Low Risk',
title.A.ylab='Risk Score',
title.B.ylab='Survival Time(year)',
title.A.legend='Risk Group',
title.B.legend='Status',
title.C.legend='Expression',
size.ABC=1.5,
size.ylab.title=12,
size.Atext=11,
size.Btext=11,
size.Ctext=11,
size.yticks=0.5,
size.yline=0.5,
size.points=2,
size.dashline=1,
size.cutoff=5,
size.legendtitle=13,
size.legendtext=12,
family='sans',
expand.x=3,
relative_heights=c(0.1,0.1,0.01,0.15)
#
)

topptx(filename = "ggrisk.pptx",width=8)
```

```
two_scatter(fit2,
            cutoff.value = 'median',
            cutoff.x = 142,
            cutoff.y = -0.5)


two_scatter(fit2,
            cutoff.value = 'median',
            cutoff.x = 142,
            cutoff.y = -0.5,
            code.0 = 'Graft Survival',
            code.1 = 'Graft Loss',
            code.highrisk = 'High Group',
            code.lowrisk = 'Low Group',
            title.A.legend = 'Riskscore',
            title.B.legend = 'Event Status',
            title.A.ylab = 'Riskscore',
            title.B.ylab = 'Survival Time(days)',
            title.xlab = 'This is rank',
            vjust.A.ylab = 1,
            vjust.B.ylab = 3,
            size.AB = 2,
            size.ylab.title = 14,
            size.xlab.title = 14,
            size.Atext = 12,
            size.Btext = 12,
            size.xtext = 12,
            size.xyticks = 0.5,
            size.xyline = 0.5,
```

```
                 size.dashline = 1.5,
                 size.points = 1,
                 size.cutoff = 5,
                 size.legendtitle = 14,
                 size.legendtext = 13,
                 color.A = c(low='green',high='red'),
                 color.B = c(code.0='green',code.1='red'),
                 family = 'sans', # sans for Arail, serif for Times New Roman
                 expand.x=10)

topptx(filename = "ggrisk2.pptx",width=8)


# DEGs between the low-risk and high-risk groups ----------------------------------------------------

library(limma)

exprset_seed126 <- GSE21374_exprset_jiaozheng[,my_seed_clini$patient]
my_seed_clini <-    read.delim("clipboard")

getDEG(exprset_seed126,141,141,"DEGs")




# GOandKEGG -------------------------------------------

###1 s

my_gene_seed156 <- read.delim("clipboard")
```

```
my_gene <- my_gene_seed156

gene <- my_gene$gene

library(clusterProfiler)
library(org.Hs.eg.db)
gene = bitr(gene, fromType="SYMBOL", toType="ENTREZID", OrgDb="org.Hs.eg.db")
class(gene)
de <- gene$ENTREZID

go <- enrichGO(gene = de, OrgDb = "org.Hs.eg.db", ont="all",pvalueCutoff = 0.05,
               qvalueCutoff = 0.05)
go_bp <- enrichGO(gene = de, OrgDb = "org.Hs.eg.db", ont="BP",pvalueCutoff = 0.05,
                  qvalueCutoff = 0.05)
beepr::beep(8)
library(ggplot2)
p <- dotplot(go, split="ONTOLOGY") +facet_grid(ONTOLOGY~., scale="free")
p
barplot(go)
p_go <- dotplot(go_bp,showCategory = 20)
p_go
barplot(go_bp,showCategory = 20)
eoffice::topptx(filename ="go_bp.pptx",width =10,
                height = 6)

gene2<- gene$ENTREZID
View(gene2)
if(!require("KEGG.db")) BiocManager::install("KEGG.db")
library("KEGG.db")
EGG <- enrichKEGG(gene= gene2,
```

```
                        organism        = 'hsa',
                        pvalueCutoff = 0.05,
                        use_internal_data = TRUE)
head(EGG)
dotplot(EGG,showCategory = 20)
barplot(EGG)
eoffice::topptx(filename ="kegg.pptx",width =10,
                      height = 6)

test1 <- data.frame(EGG)
test2 <- data.frame(go_bp)

write.xlsx(test1,file = "kegg.xlsx")
write.xlsx(test2,file = "go_bp.xlsx")



# GSEA ----------------------------------------------



library(clusterProfiler)
library(org.Hs.eg.db)
library(enrichplot)


library(clusterProfiler)
library(org.Hs.eg.db)
library(enrichplot)
myGSEA <- read.delim("clipboard",header = TRUE,row.names = 1)

myGSEA$SYMBOL <- row.names(myGSEA)
myGSEA <- myGSEA %>% dplyr::select(SYMBOL,everything())
```

```
input <- myGSEA

gene_df <- bitr(input$SYMBOL,
                fromType = "SYMBOL",
                toType = "ENTREZID",
                OrgDb = org.Hs.eg.db)

gsea_data <- merge(input,
                   gene_df,
                   by = "SYMBOL",
                   all = FALSE,
                   sort = FALSE)

summary(gsea_data)

gsea_data_order <- gsea_data[order(gsea_data$logFC, decreasing = T),]
gsea_data_order <- gsea_data_order[is.na(gsea_data_order[,"ENTREZID"])==F,]
head(gsea_data_order)

gene.expr = gsea_data_order$logFC
names(gene.expr) <- gsea_data_order$ENTREZID
head(gene.expr)
#save(gene.expr,file = "GSEA.Rdata")

kk <- gseKEGG(gene.expr, organism = "hsa",pvalueCutoff = 0.05)
beepr::beep(8)
head(kk)
dim(kk)
save(kk,file = "GSEA.Rdata")
```

```
sortkk<-kk[order(kk$enrichmentScore, decreasing = T),]
head(sortkk)
dim(sortkk)
library(openxlsx)
write.xlsx(sortkk,file = "GSEA.xlsx",rowNames=TRUE)
library(enrichplot)
paths <- read.delim("clipboard")[,1]
path <- paths[1:2]
gseaplot2(kk, path, subplots=c(1,3))

# ROC --------------------------------------------------




library(survival)
library(survminer)
library(timeROC)
names(riskresult_duoyinsu)
riskresult_duoyinsu$time <- riskresult_duoyinsu$time/365
riskresult_seed156$time <- riskresult_seed156$time/365

bioROC2=function(inputFile=null, rocFile=null){

  rt=inputFile
  #ROC
  ROC_rt=timeROC(T=rt$time,delta=rt$status,
                 marker=rt$riskscore,cause=1,
                 weighting='aalen',
                 times=c(1,3),ROC=TRUE)
```

```
  pdf(file=rocFile,width=5,height=5)
  plot(ROC_rt,time=1,col='green',title=FALSE,lwd=2)
  plot(ROC_rt,time=3,col='blue',add=TRUE,title=FALSE,lwd=2)
  legend('bottomright',
         c(paste0('AUC at 1 years: ',sprintf("%.03f",ROC_rt$AUC[1])),
           paste0('AUC at 3 years: ',sprintf("%.03f",ROC_rt$AUC[2]))
         ),
         col=c("green",'blue'),lwd=2,bty = 'n')
  dev.off()
}

bioROC2(inputFile=riskresult_seed156, rocFile="ROC1.ROC.pdf")




# Riskscore and immune cell------------------------------------------------------

names(riskresult_duoyinsu)
names(my_data)

seed156_jin <- my_data %>% left_join(riskresult_seed156,by="patient") %>%
  column_to_rownames(var = "patient") %>% select(riskscore,everything()) %>%
  select(1:23)
names(seed156_jin)
exprSet <- seed156_jin
colnames(exprSet)
str(exprSet)
y <- as.numeric(exprSet[,1])
colnames <- colnames(exprSet)
```

```
cor_data_df <- data.frame(colnames)
for (i in 1:length(colnames)){
  test <- cor.test(as.numeric(exprSet[,i]),y,type="spearman")
  cor_data_df[i,2] <- test$estimate
  cor_data_df[i,3] <- test$p.value
}
names(cor_data_df) <- c("symbol","correlation","pvalue")
head(cor_data_df)
write.xlsx(cor_data_df,file = "cor.xlsx")

write_clip(cor_data_df)



data <- read.delim("clipboard")

print(colnames(data))
data=data[order(data$cor),]
data$immuneGene <- factor(data$immuneGene,levels=data$immuneGene)
data$abs_cor=abs(data$cor)
data
library(ggplot2)
p=ggplot(data, aes(x=immuneGene, y=cor))+
  geom_col( show.legend = F,width = 0.05)+
  geom_point(aes(size=abs_cor,col=pvalue))+
  coord_flip()+
  theme_light()+
  scale_color_viridis_c()

p+annotate("text",x=data$immuneGene,y=1.1,label= round(data$pvalue,6),hjust=1)
p1 <- p+annotate("text",x=data$immuneGene,y=1.1,label= round(data$pvalue,6),hjust=1)
```

```
p1+xlab(label = "immunecell")

topptx(filename = "seed156.pptx")
```
