# Supplementary material for: Immune-Related Genes for Predicting Future Kidney Graft Loss: A Study Based on GEO Database
Source: Front Immunol. 2022 Feb 25;13:859693. doi: 10.3389/fimmu.2022.859693 (PMC8913884; doi:10.3389/fimmu.2022.859693)
Supplement: Supplementary file 1 [file DataSheet_1.zip › Supplementary material/Supplementary Table 1.DOCX]

Supplementary Table 1. Univariate Cox analysis results of feature genes

| Gene | HR(95%CI) | P value |
| --- | --- | --- |
| CXCL11 | 1.340 (1.190-1.509) | <0.001 |
| CXCL10 | 1.375 (1.175-1.610) | <0.001 |
| CCL4 | 1. 751(1.423-2.155) | <0.001 |
| IDO1 | 1.355 (1.157-1.588) | <0.001 |
| GBP2 | 1.969 (1.545-2.510) | <0.001 |

HR, Hazard Ratio; 95%CI, 95% confidence interval.
