# Supplementary figures and images for: Immune-Related Genes for Predicting Future Kidney Graft Loss: A Study Based on GEO Database
Source: Front Immunol. 2022 Feb 25;13:859693. doi: 10.3389/fimmu.2022.859693 (PMC8913884; doi:10.3389/fimmu.2022.859693)

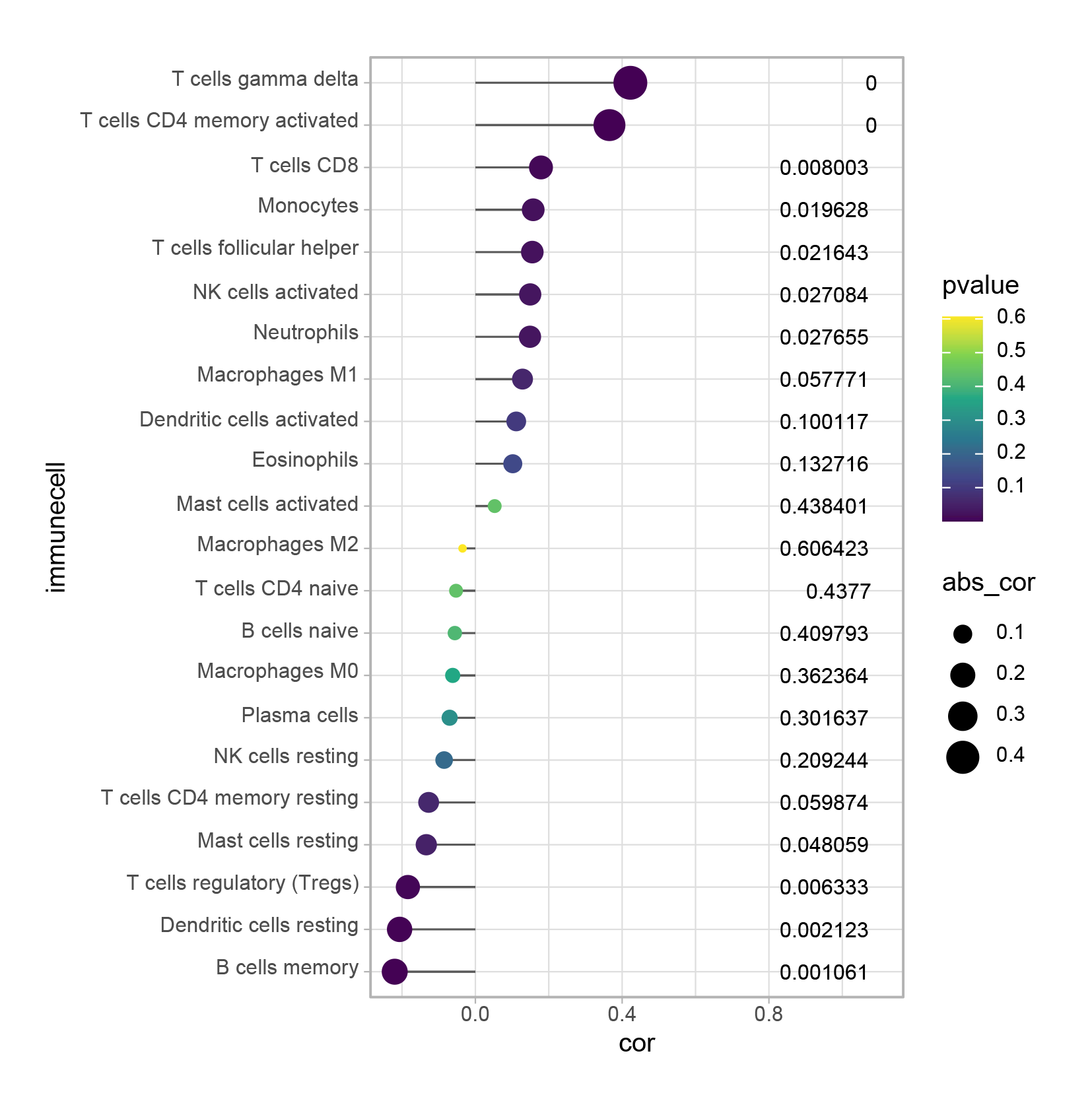

Supplement: Supplementary file 1 [file DataSheet_1.zip › Supplementary material/Supplementary Figure 1.TIF]
